# PMDS-YOLO: A Lightweight Deep Learning Model for Non-Destructive Citrus Fruit Diameter Estimation and Quality Grading

**DOI:** 10.3390/foods15183211

**Published:** 2026-09-11

**Authors:** Hang Liu, Zhiyong Cao, Zifei Ma, Binyuan Zhong, Siyu Li, Yulin Li, Aiying Yin, Ziyi Yang, Rong Liu, Zhijia Wang, Jihong Sun, Tong Li

**Affiliations:** 1 College of Big Data, Yunnan Agricultural University, Kunming 650201, China; 15388895367@163.com (H.L.);; 2 College of Water Resources, Yunnan Agricultural University, Kunming 650201, China; 3 College of Agronomy and Biotechnology, Yunnan Agricultural University, Kunming 650201, China; 4 The Key Laboratory for Crop Production and Smart Agriculture of Yunnan Province, Yunnan Agricultural University, Kunming 650201, China; 5 School of Nursing, Yunnan University of Chinese Medicine, Kunming 650500, China; 6 College of Architecture and Engineering, Yunnan Agricultural University, Kunming 650201, China; 7 School of Information Engineering, Kunming University, Kunming 650214, China; 8 Yunnan Key Laboratory of Intelligent Logistics Equipment and Systems, Kunming 650214, China

**Keywords:** citrus fruit, diameter estimation, quality grading, deep learning, edge deployment

## Abstract

Citrus fruit size is a core quality attribute that determines commercial value and market acceptance. In postharvest processing, rapid, accurate, and non-destructive diameter estimation is an essential prerequisite for automated grading, yet existing computer vision methods are difficult to deploy on factory edge devices. To address this problem, this paper proposes PMDS-YOLO, a lightweight deep learning model for non-destructive citrus fruit diameter estimation and automated quality grading. The model incorporates four key improvements: width factor compression to 0.125 to eliminate channel redundancy; adoption of a MobileOne backbone embedded with the proposed multi-scale channel–spatial residual attention module (MCSR), which reduces parameter count while enhancing multi-scale feature extraction and background discrimination; introduction of dynamic snake convolution (DySnakeConv) to improve the feature extraction module for better detection of irregular fruit contours; and adoption of soft non-maximum suppression (Soft-NMS) to reduce missed detections of densely overlapping targets. Experimental results on a self-built citrus dataset show that PMDS-YOLO achieves only 0.58 M parameters and 86.41% mAP@0.5:0.95, representing a 76.4% reduction in parameter count and a 0.99 percentage point improvement in mAP over the baseline YOLOv13n. In the diameter estimation stage, a quadratic polynomial regression calibration model reduces the MAE to 2.15 mm (R^2^ = 0.85, RMSE = 3.16 mm) and achieves an overall grading accuracy of 57.0% in the grading evaluation, indicating that while the method demonstrates potential in diameter estimation accuracy, further optimization is required for grading performance. The proposed system provides a low-cost, edge-deployable solution for preliminary fruit size assessment and small-scale sorting applications.

## 1. Introduction

Citrus is one of the most widely planted economic fruit trees globally and is also a key supported industry for promoting rural revitalization in China [1]. Citrus fruits are rich in vitamin C, flavonoids, dietary fiber, and various minerals, possessing multiple health benefits such as antioxidant, anti-inflammatory, and digestive-promoting effects [2]. In the food industry, fruit size is one of the most intuitive quality attributes directly influencing consumer preference, commercial value, and market price [3]. Fruit diameter, in particular, serves as a core indicator for citrus quality evaluation, grading, and sorting, as well as for yield estimation. Traditional manual measurement is time-consuming, labor-intensive, and prone to causing scratches on the fruit epidermis, which compromises postharvest quality and reduces shelf life. Therefore, achieving rapid, accurate, and non-destructive estimation of citrus fruit diameter is of critical importance for quality assurance in postharvest processing and automated grading systems.

Existing vision-based fruit diameter estimation methods are primarily categorized into two types: traditional methods based on 2D/3D geometric measurement, and deep learning-based detection and estimation methods. Geometric measurement-based methods have achieved significant progress. Kim et al. [4] relied on 3D point cloud data acquired by LiDAR, screening boundary points using a Gaussian mixture model and reconstructing the contour perimeter to calculate diameter at breast height. Yan et al. [5] used a depth camera to obtain apple depth information and the diagonal length of the bounding rectangle in 2D images, constructing a diameter estimation model combined with multiple regression. Kuzelka et al. [6] employed a handheld laser scanner to acquire tree point clouds and achieved unbiased diameter at breast height estimation by optimizing the RANSAC algorithm. Prabhu et al. [7] utilized UAV-borne LiDAR combined with deep learning methods to achieve diameter profile estimation. Although these methods have achieved promising results, they rely heavily on specialized equipment such as LiDAR and depth cameras, resulting in high deployment costs, poor environmental adaptability, and susceptibility to interference, which in turn causes issues including missing point cloud data and incomplete contour reconstruction. These limitations make it difficult to meet the deployment requirements of low-cost, on-site food processing applications, particularly in small- and medium-sized citrus processing facilities where equipment budgets are constrained.

In recent years, the rapid development of deep learning has provided a new technological pathway for fruit diameter estimation [8]. By selecting an appropriate object detection model, quantitative estimation of fruit diameter can be efficiently accomplished based on accurate fruit localization. Current object detection models based on convolutional neural networks (CNNs) are mainly divided into two categories. One category is the region proposal-based two-stage detection algorithms, represented by Region-based Convolutional Neural Network (R-CNN) [9], Fast R-CNN [10], and Faster R-CNN [11]. These methods first extract candidate target regions and then perform classification and regression localization based on region features [12]. In agricultural and food detection tasks, Cui et al. [13] proposed a weed identification method at the soybean seedling stage based on UAV images and Faster R-CNN, achieving an average detection accuracy of 88.69%. Zhang et al. [14] proposed a soybean leaf disease detection method based on multi-feature fusion Faster R-CNN, achieving an average detection accuracy of 83.34%. Quan et al. [15] proposed an improved Faster R-CNN-based maize seedling detection method, achieving a detection accuracy of 97.71% for maize seedlings and weeds in complex field environments. Tu et al. [16] proposed a passion fruit detection and counting method based on multi-scale Faster R-CNN, achieving a detection accuracy of 96.20%. Although two-stage methods excel in detection accuracy, the decoupling of candidate region generation and classification/regression stages incurs substantial inference redundancy, slow speed, and high parameter and computational costs, making them unsuitable for edge deployment in real-time food grading lines.

The other category is regression-based single-stage detection algorithms, with typical representatives including the You Only Look Once (YOLO) series [17], Single Shot MultiBox Detector (SSD) [18], and Retina Network (RetinaNet) [19]. These methods do not require a candidate region generation step and directly perform dense category prediction and bounding box regression on the image. Liu et al. [20] proposed a tea bud detection method based on YOLOv8 (YOLO-TBD), achieving an average detection accuracy of 87.04% with 41.27 M parameters. Guo et al. [21] proposed a cotton leaf disease detection method based on an improved SSD, achieving an average detection accuracy (mAP) of 84.80% with a parameter size of 23.16 MB. Guo et al. [22] proposed a weed detection method for sugar beet fields based on an enhanced RetinaNet and contextual semantic fusion (WeedNet-R), achieving an average detection accuracy (mAP) of 92.30% with 38.0 M parameters. Although single-stage models are significantly superior to two-stage methods in terms of detection speed and parameter count, their size and computational overhead remain substantial in edge deployment scenarios with severely limited computing power and storage resources, making it difficult to meet the real-time detection requirements of high-throughput food processing lines.

To address the above issues, researchers have recently conducted extensive work on the lightweight design of single-stage detection models and have made notable progress in citrus detection tasks. Lu et al. [23] proposed Yolo-FD, which embeds 3D coordinate attention in the backbone network, adopts a simplified bidirectional feature pyramid network, and introduces context transformation (COT) in the neck, achieving an mAP@0.5 of 98.7% on a self-built citrus dataset with 6.25 M parameters. Chen et al. [24] proposed FEW-YOLOv8n, which introduces the FasterNet Block into the C2f module to reduce parameter count and computational cost, and embeds efficient multi-scale attention (EMA) and the Wise-IoU loss function, achieving an mAP of 91.7% with 2.69 M parameters. Liao et al. [25] proposed YOLO-MECD, which introduces the EMA mechanism, a partial convolution-based CSPPC module, and the MPDIoU loss function, achieving an mAP of 81.6% with 2.30 M parameters. Gu et al. [26] proposed YOLO-DCA, which adopts depthwise separable convolution, coordinate attention-fused convolution, and a dynamic detection head, achieving an mAP of 96.98% with 2.28 M parameters. Although the above studies have compressed the parameter count to the megabyte level, the existing lightweight design paradigm largely focuses on parameter reduction, with limited attention to the collaborative optimization of computational cost and latency, while some modules still exhibit structural redundancy. Therefore, achieving a favorable balance among model size, inference speed, and detection accuracy remains a key challenge for edge deployment in food quality assessment applications. To tackle these challenges, this study proposes PMDS-YOLO, a lightweight object detection model built upon an improved YOLOv13n, aiming to provide an efficient, non-destructive, and edge-deployable solution for citrus fruit quality assessment and automated postharvest grading.

The main contributions of this paper are summarized as follows:

1. Dataset construction. A citrus image dataset covering both orchard and laboratory scenarios was constructed, containing diverse fruit characteristics such as complex lighting, multi-level occlusion, multi-angle shooting, and multi-scale variations.
2. A lightweight citrus detection model, PMDS-YOLO, oriented towards edge deployment, is proposed. Using YOLOv13n as the baseline, four improvements are implemented: width factor compression, MCSR-MobileOne backbone reconstruction, Dynamic Snake Convolution (DySnakeConv) module optimization, and Soft-Non-Maximum Suppression (Soft-NMS) post-processing. As a result, the parameter count is reduced by 76.4%, the model size is compressed by 72.3%, while the mAP is improved by 0.99 percentage points and the precision is improved by 2.08 percentage points.
3. A Multi-scale Channel-Space Residual attention module (MCSR) is proposed. This module first constructs a hierarchical receptive field through cascaded multi-branch depthwise dilated convolutions (DDConv) to extract multi-scale contextual features. Subsequently, it serially connects lightweight Efficient Channel Attention (ECA) and Spatial Attention (SA) to achieve channel-adaptive weighting and fruit region response enhancement, effectively suppressing background interference. Finally, residual connections are employed to fuse the original and enhanced features, ensuring training stability and feature representation capability. Overall, this module significantly improves detection accuracy with extremely low parameter overhead.
4. A low-cost, high-precision, and standardized fruit diameter estimation and grading system is established. Using a standard coin as a reference object, this system integrates object detection, non-contact initial diameter measurement based on the principle of similar triangles, systematic error calibration based on quadratic polynomial regression, and intelligent grading according to citrus industry standards, realizing an integrated pipeline of “object detection–diameter estimation–intelligent grading” for food quality assessment.
5. The proposed PMDS-YOLO model is efficiently deployed to Android edge devices. On this basis, the system achieves integrated operations of real-time fruit detection, diameter calculation, error calibration, and grade determination in offline environments, providing a lightweight, low-power, and easily deployable intelligent solution for real-time citrus field detection and factory automated sorting.

## 2. Materials and Methods

**Figure 1:**
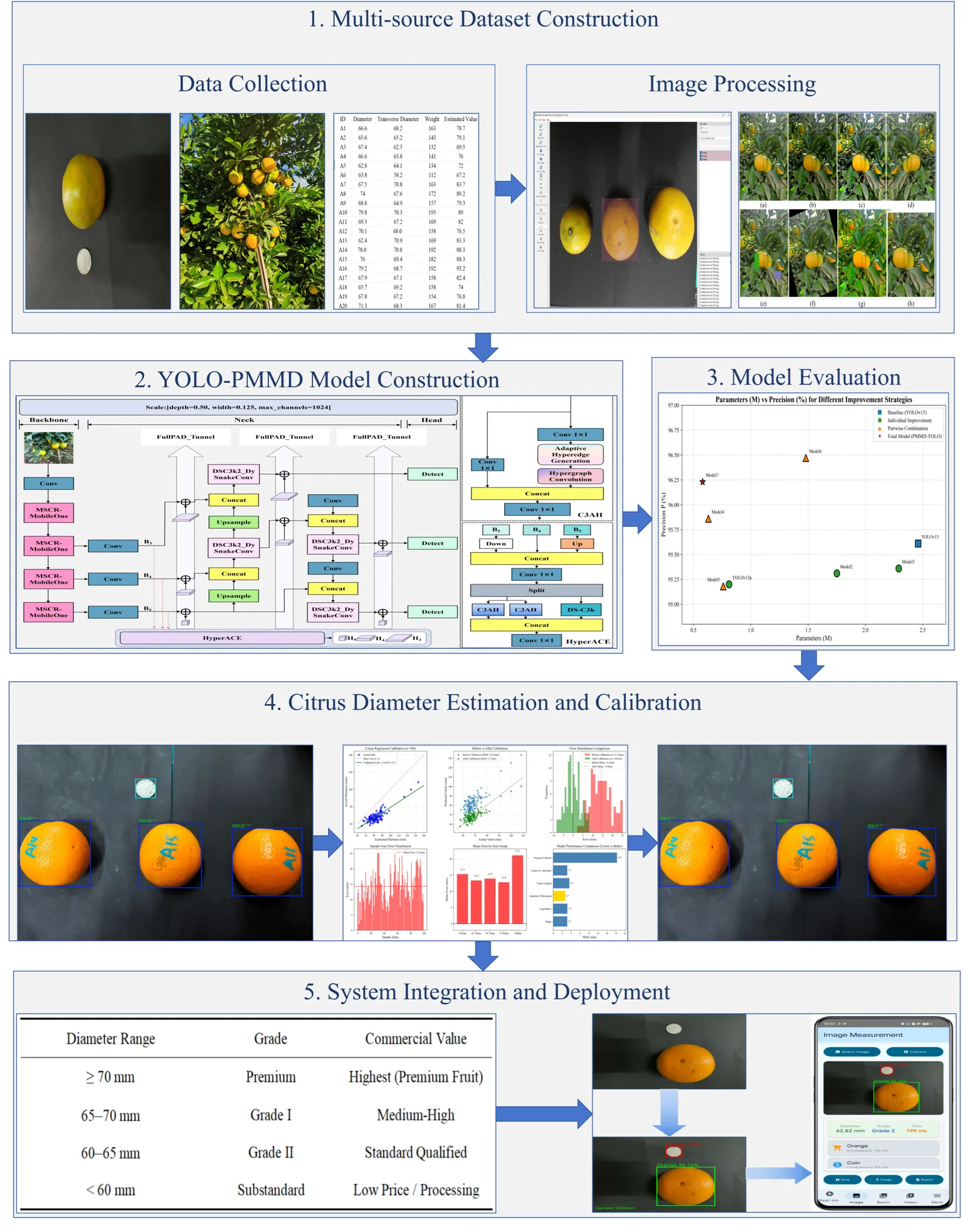
Research technology roadmap.

In this study, a OnePlus Ace5 smartphone(OnePlus, Shenzhen, China) was used to capture original images of citrus fruits in an orchard. The fruits were then brought back to the laboratory, where images of coins and citrus fruits in the same frame were collected. Meanwhile, a vernier caliper and an electronic balance were used to measure the true longitudinal and transverse diameters of the citrus fruits, respectively, providing data support for subsequent model training and diameter calibration. A multi-dimensional optimization was performed based on the YOLOv13n architecture to construct the PMDS-YOLO lightweight object detection model suitable for citrus detection scenarios. To validate the effectiveness of PMDS-YOLO, multi-dimensional comparative analyses were conducted against multiple mainstream models and ablation models on a self-built citrus dataset. In the diameter estimation stage, PMDS-YOLO was first used to detect the pixel diameters of the bounding boxes of the coin and the fruit, and the actual fruit diameter was preliminarily derived based on the principle of similar triangles. Second, a quadratic polynomial regression model was constructed using the derived values as inputs and the measured values as outputs to calibrate systematic bias. Finally, automated fruit grading was accomplished in accordance with citrus industry size grading standards. Based on the above methods, this study developed a citrus diameter estimation and size grading system and deployed it on Android edge devices, achieving real-time fruit detection, diameter estimation, and grade determination. The overall technical workflow is shown in Figure 1.

### 2.1. Data Preparation

#### 2.1.1. Study Area

**Figure 2:**
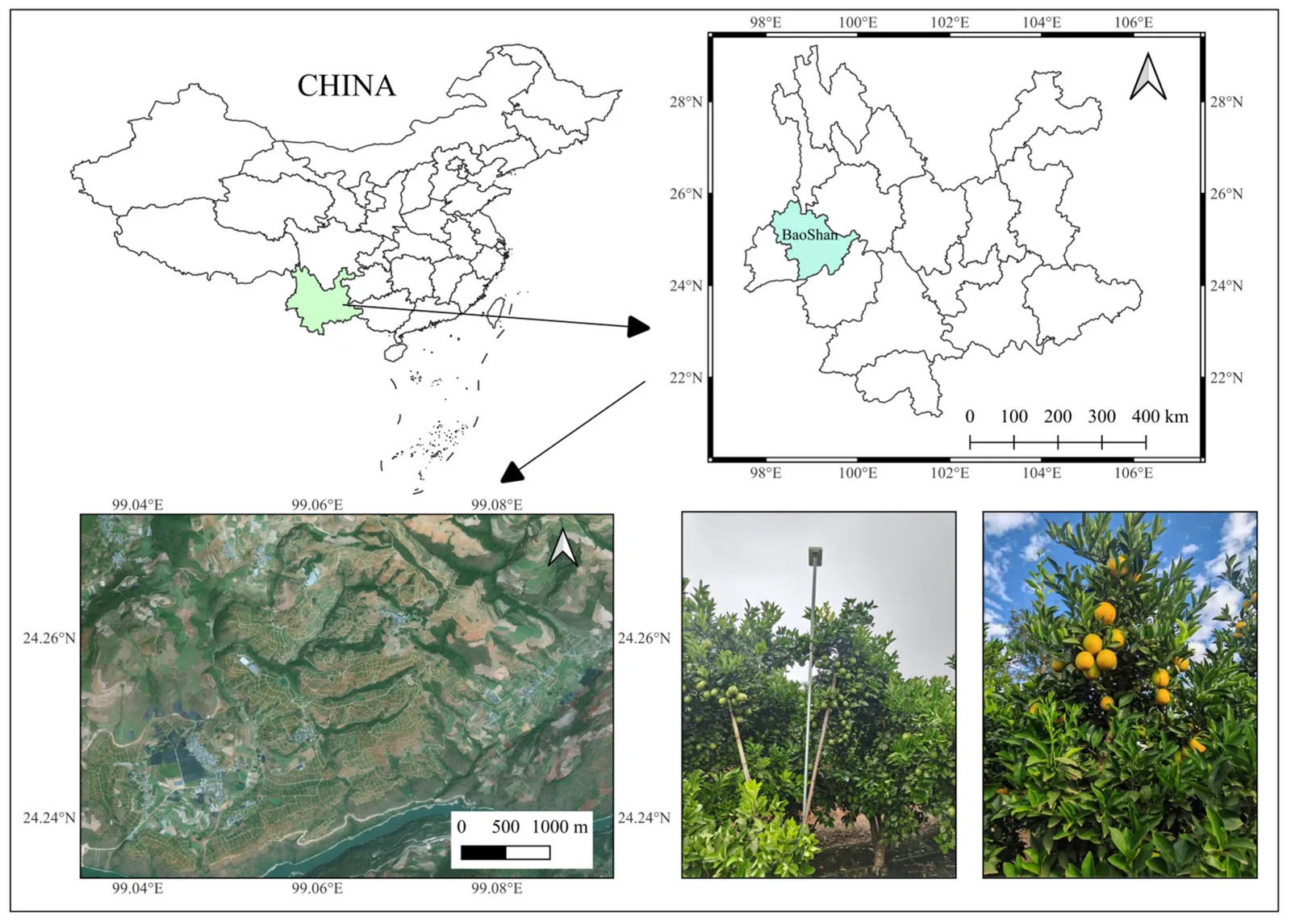
Geographic location of the study area.

As shown in Figure 2, the experimental site is located at the Chu Orange Base in Longling County, Baoshan City, Yunnan Province, China (99°3.61′ E, 24°14.75′ N), situated in the Nujiang River Valley. This area features a southern subtropical monsoon climate, with an annual average temperature of 20.06 °C and an annual precipitation of approximately 800 mm. The climate is warm and moderate without severe cold or frost. Coupled with the favorable drainage and ventilation conditions of the river valley, this environment provides excellent light, heat, water, and soil conditions for citrus cultivation.

#### 2.1.2. Data Acquisition

**Figure 3:**
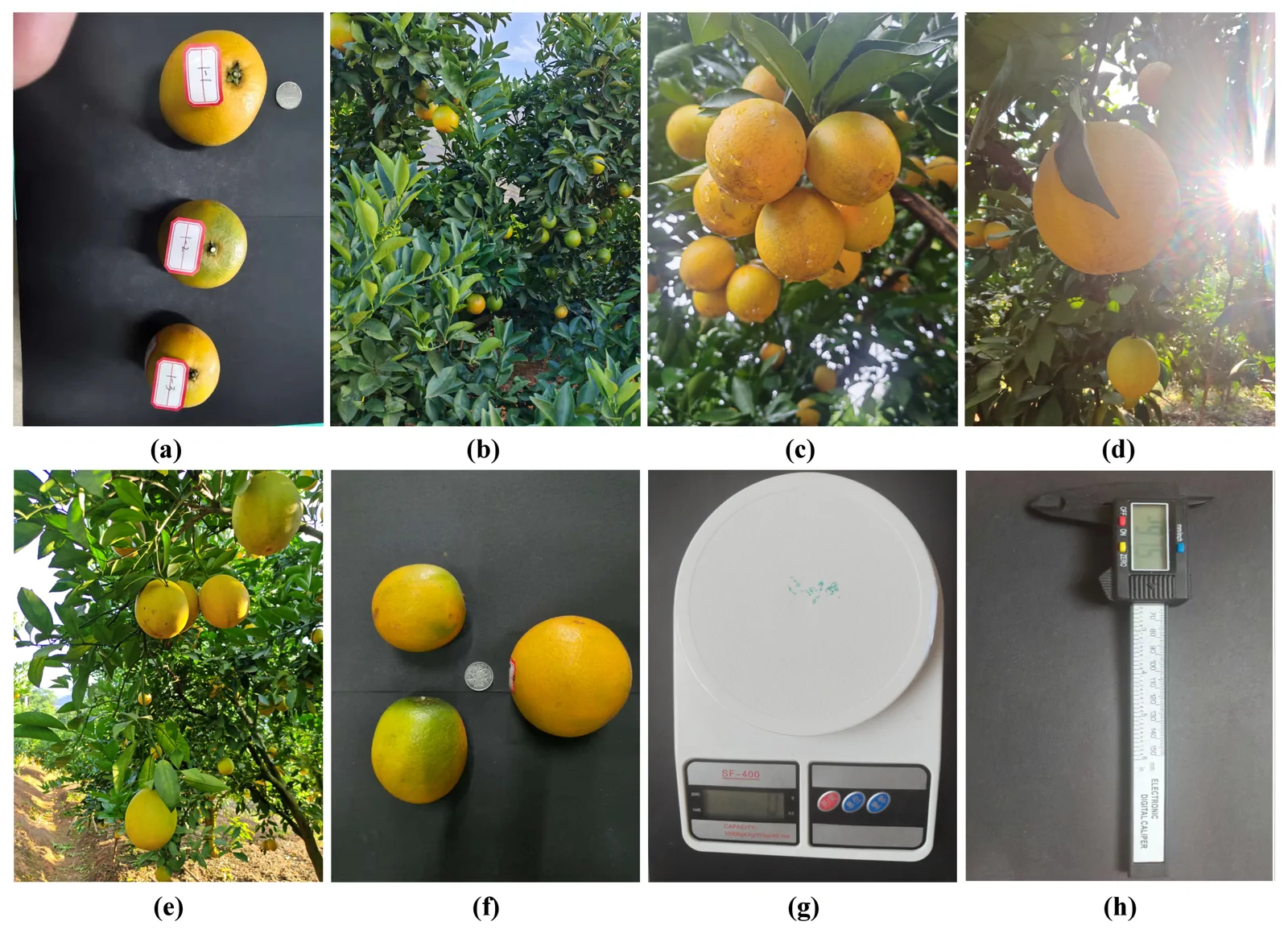
Schematic diagram of data collection and sample characteristics. (**a**) Multi-scale; (**b**) Different ripeness; (**c**) Dense distribution; (**d**) Strong sunlight; (**e**) Orchard scene; (**f**) Laboratory scene; (**g**) Electronic balance; (**h**) Vernier caliper.

In November 2025, citrus fruit images were acquired using a OnePlus Ace5 smartphone (1919 × 1080 resolution) in both orchard and laboratory settings, captured at horizontal, top-down, and oblique-side angles and at distances ranging from 10 cm to 120 cm. A total of 1344 original images were acquired, covering diverse scenarios including varying illumination, occlusion, fruit density, and maturity levels (Figure 3). Image acquisition prioritized scene diversity over systematic multi-angle repeated captures of fixed fruit individuals; the vast majority of images originated from distinct fruit specimens, with only a negligible fraction incidentally captured multiple times. The 100 fruits brought to the laboratory were each photographed once under controlled conditions and assigned to the validation and test sets. During dataset partitioning, all images of the same fruit were assigned to the same subset, ensuring that no homologous images appeared across the training, validation, and test sets, thereby guaranteeing sample independence. Concurrently, the longitudinal diameter (from the calyx to the apex) and the transverse diameter (the widest dimension) of the 100 fruits were measured using a vernier caliper, and single-fruit weight was measured using an electronic balance. The transverse diameter is the designated grading dimension according to the DB5304/T 003-2019 standard. Although the height of the fruit bounding box corresponds to the longitudinal direction, the strong correlation between the longitudinal and transverse diameters supports the use of the measured transverse diameter as the reference value for regression calibration, establishing a mapping from the image-derived estimates to the transverse diameter for direct application to grade determination.

#### 2.1.3. Data Preprocessing

**Figure 4:**
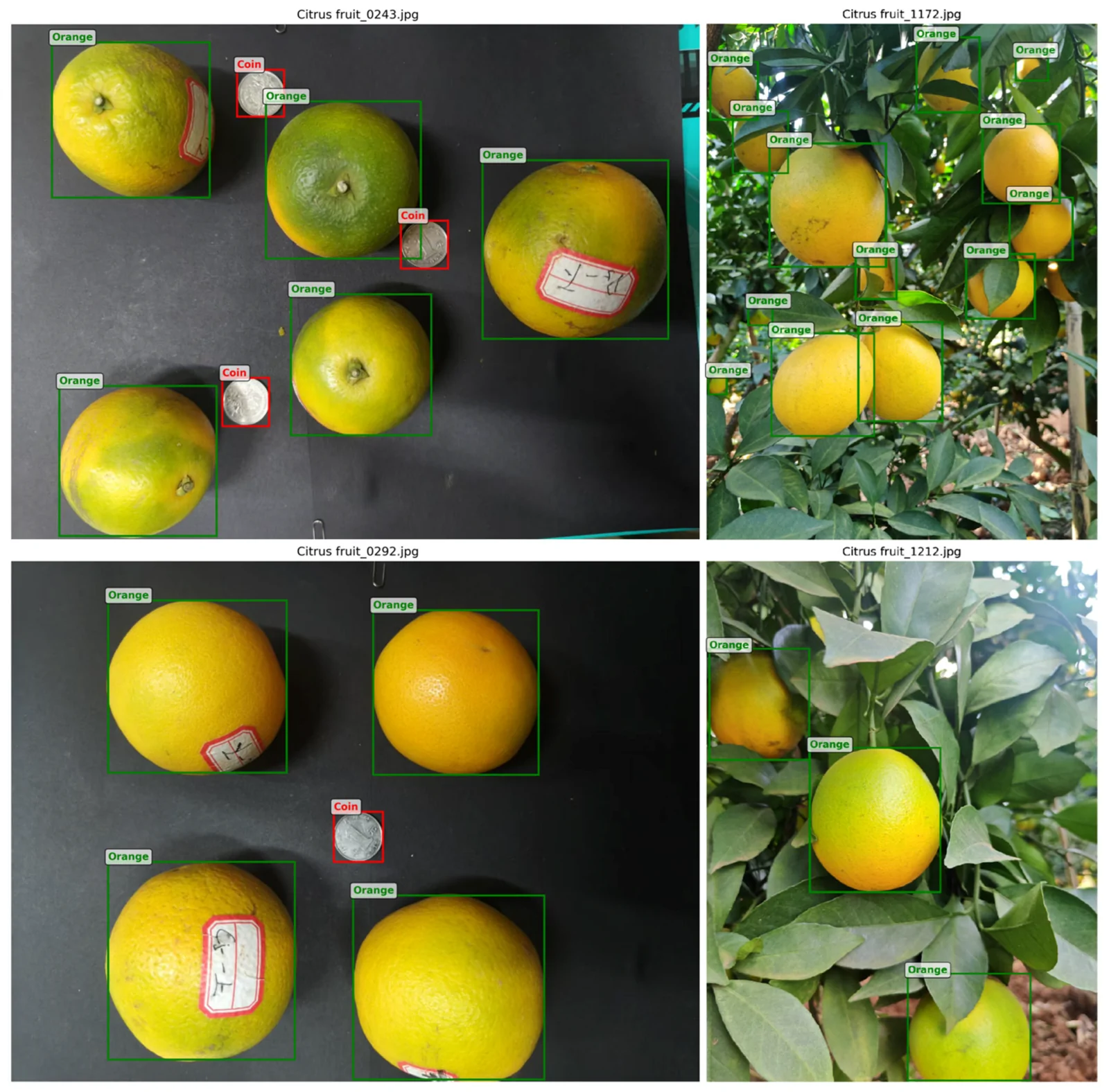
Example of data annotation.

**Figure 5:**
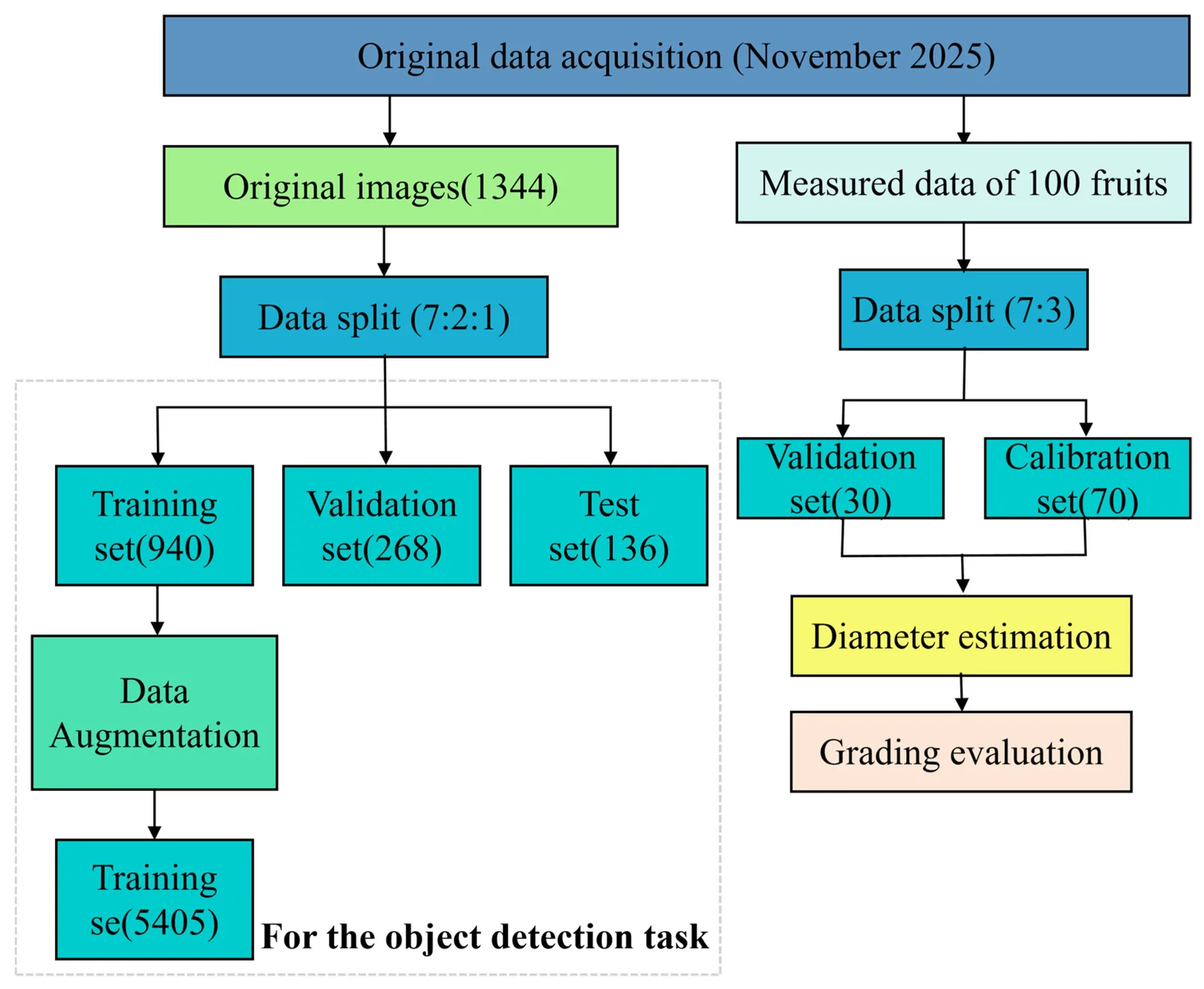
Schematic illustration of the dataset partitioning strategy.

In this study, LabelImg (v1.8.6) was used to annotate citrus fruits and coins in the images using bounding rectangles, with labels designated as “Orange” and “Coin”, respectively. For occluded fruits, annotations were performed based on their actual visible regions. The annotation results were saved as TXT files in the YOLO format, with file names kept consistent with the corresponding image names, as shown in Figure 4. Concurrently, the measured longitudinal diameter and single-fruit weight data underwent data cleaning, with missing values imputed using the mean, and outliers directly removed, thereby ensuring data quality for subsequent regression model training. Figure 5 illustrates the complete dataset partitioning strategy. The workflow comprises three components: (1) Object detection task: the 1344 original images are randomly divided into training (70%), validation (20%), and test (10%) sets at a ratio of 7:2:1; (2) Diameter calibration task: the 100 laboratory-measured fruits are split into a calibration set (70 fruits) for fitting the quadratic polynomial regression and an independent validation set (30 fruits) for evaluating estimation accuracy; (3) Grading assessment: all 100 fruits are used for grading accuracy evaluation. Notably, while the test set for object detection contains images of these 100 fruits, the regression calibration and validation are based exclusively on their measured ground-truth diameters, ensuring that the samples used for grading evaluation are independent of the calibration procedure.

#### 2.1.4. Data Augmentation

**Figure 6:**
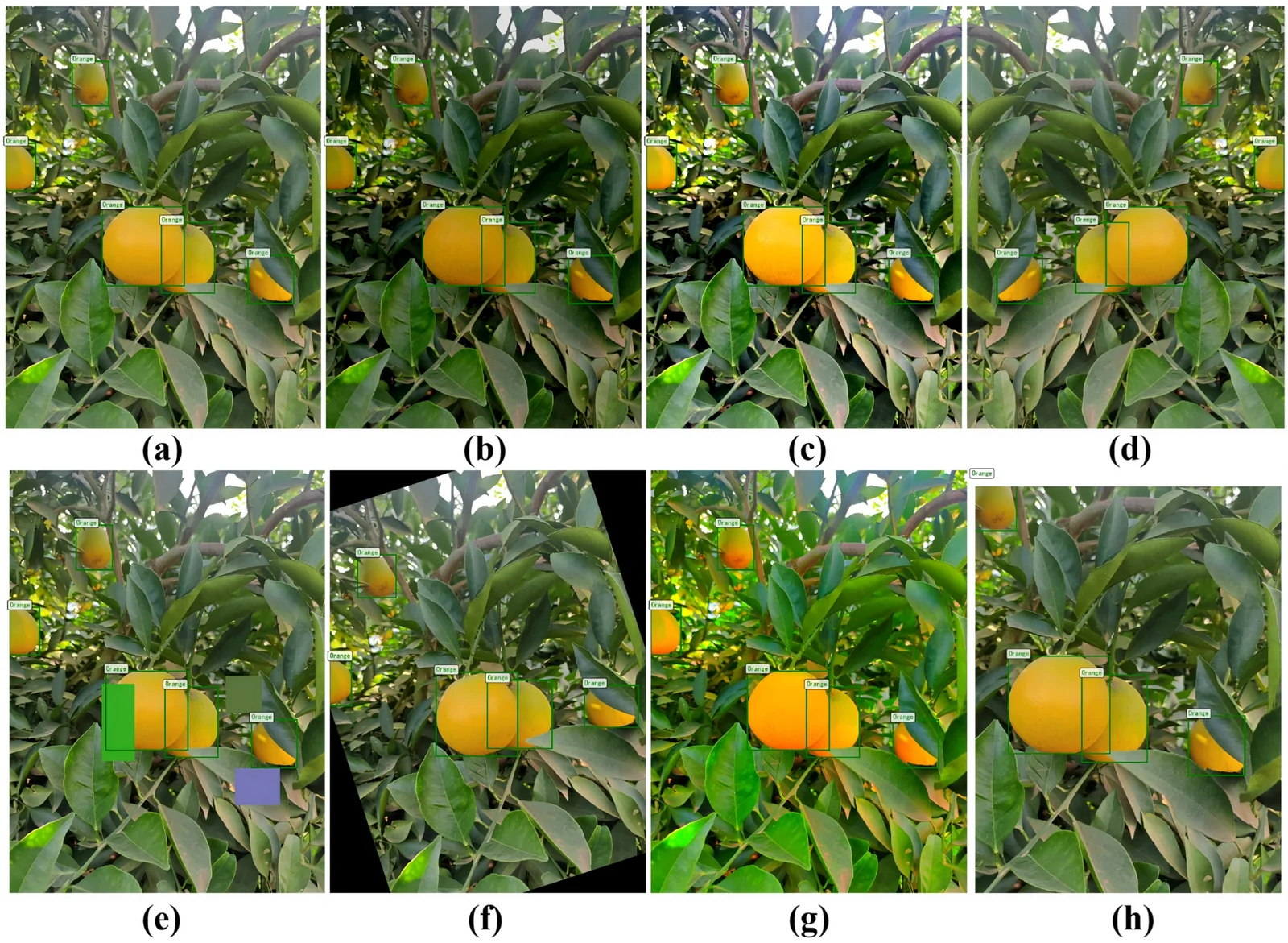
Examples of data augmentation effects: (**a**) Original image; (**b**) Brightness adjustment; (**c**) Contrast adjustment; (**d**) Horizontal flip; (**e**) Random occlusion, where the green and purple rectangular boxes represent random occlusion masks applied to simulate branch and leaf occlusion; (**f**) Random rotation; (**g**) Saturation adjustment; (**h**) Random scale.

**Figure 7:**
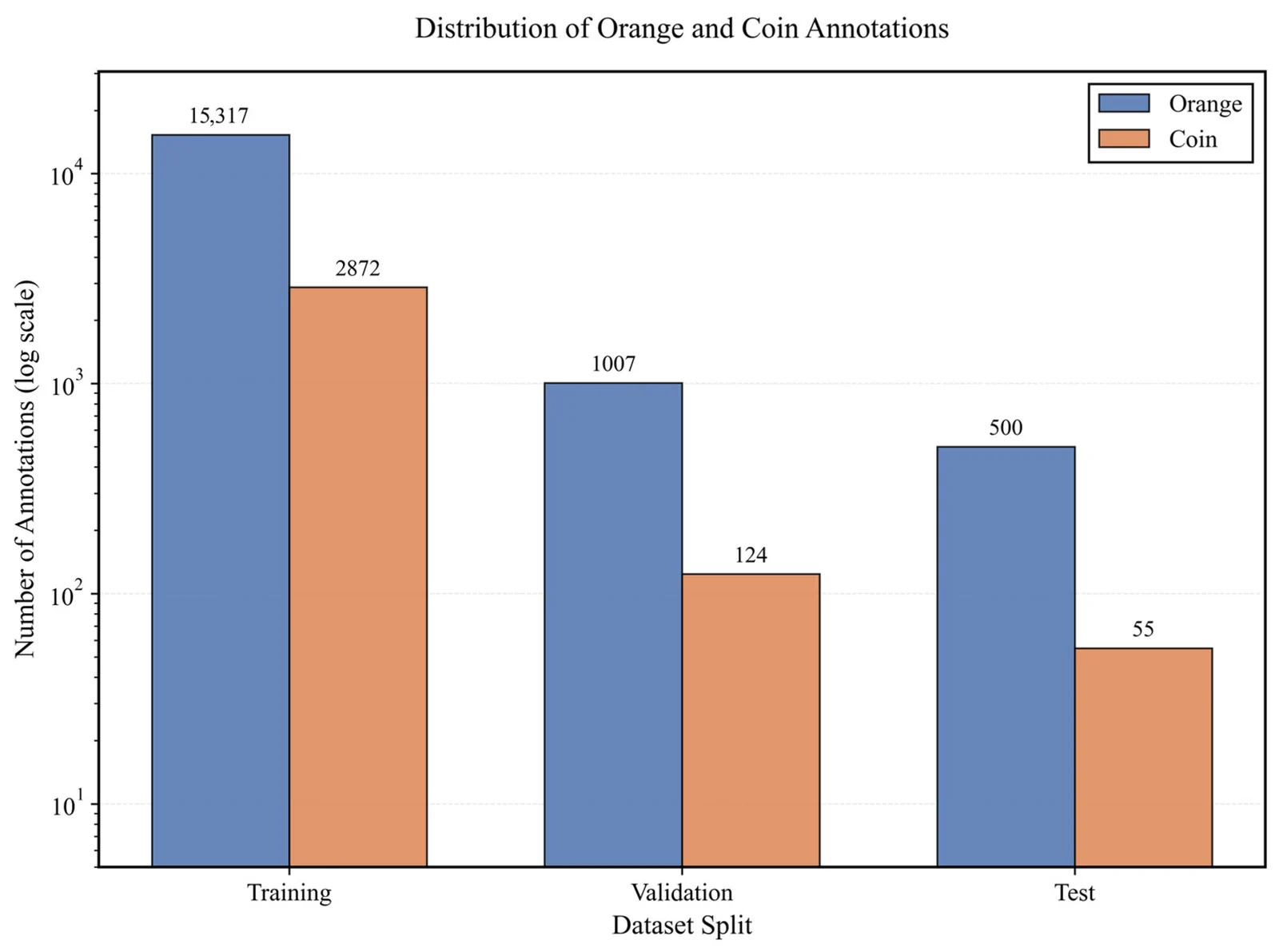
Dataset split statistics.

Given the limited size of the original training set, which precludes comprehensive coverage of the full spectrum of phenotypic variations in complex field environments, systematic data augmentation was applied to the training set to simulate the diverse variations in illumination, orientation, scale, and occlusion under natural citrus growth conditions, while the validation and test sets were kept in their original form to ensure objective evaluation of model generalization performance. The augmentation operations were configured with the following parameters: random horizontal flipping (probability 0.7); random scaling (0.5–1.8×); random rotation (±35°); random occlusion (3 rectangular masks, each covering 5–20% of the image area, colored in green or blue to simulate branch and leaf occlusion); and brightness (–0.6 to +0.5), contrast (0.2–1.5×), and saturation (0.1–3.0×) adjustments to simulate variations in light intensity, ambient brightness, and fruit maturity. All augmentation operations were randomly applied with probabilities ranging from 80% to 90%, with bounding box labels synchronously updated to ensure precise alignment between annotations and transformed images. Representative examples of the original image and augmented samples are shown in Figure 6. Following augmentation, the training set was expanded from 940 to 5405 images, while the validation and test sets remained at their original sizes of 268 and 136 images, respectively. The dataset split proportions are illustrated in Figure 7, which presents the annotated instance counts for each category across the training, validation, and test sets.

### 2.2. Construction of the Novel PMDS-YOLO Network

**Figure 8:**
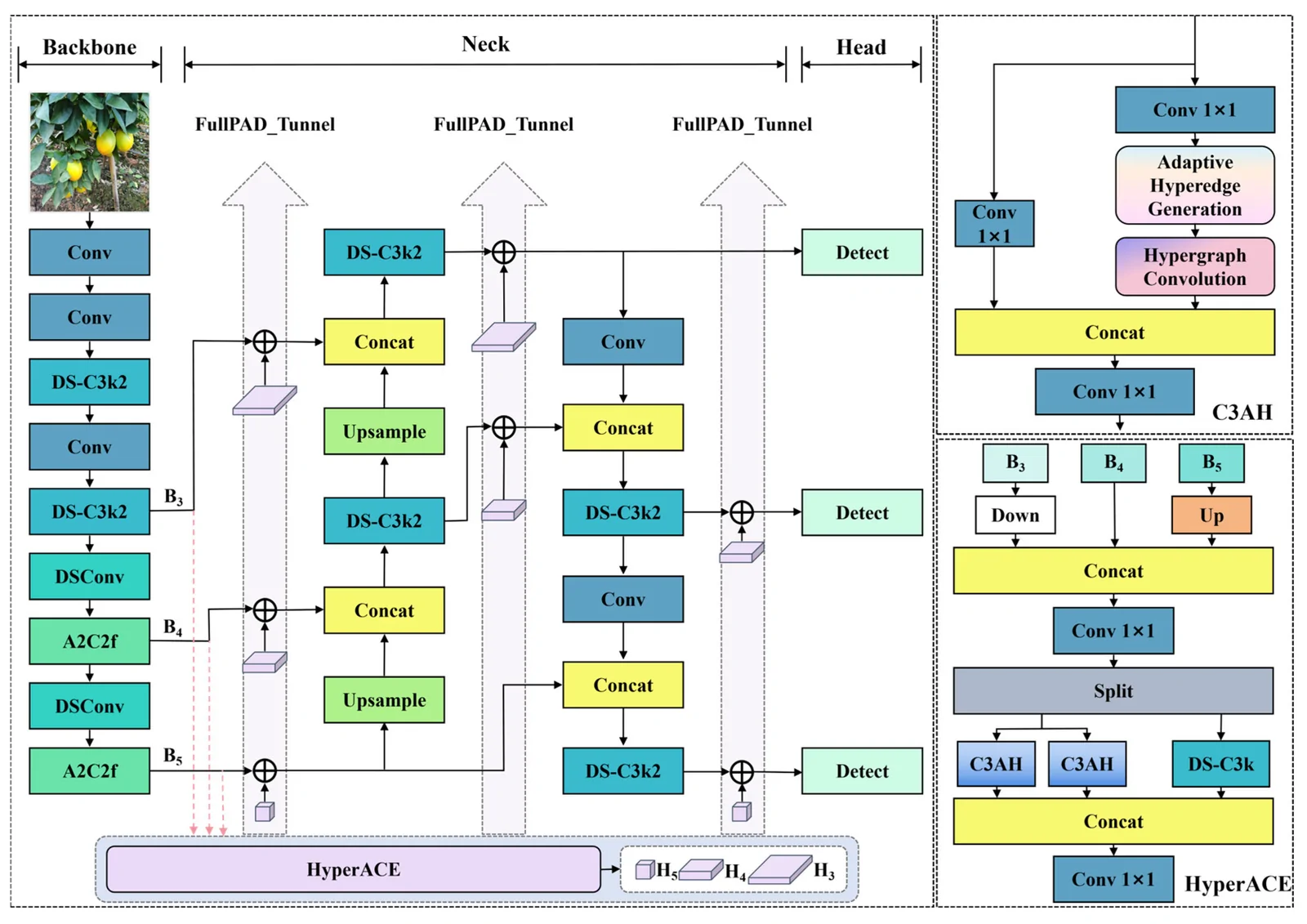
Schematic diagram of the YOLOv13 model architecture.

YOLOv13 [27] is a lightweight real-time object detector proposed by Tsinghua University, which improves upon YOLOv12 [28] by incorporating adaptive hypergraph computation into the YOLO architecture. This method designs a Hypergraph Adaptive Correlation Enhancement (HyperACE) mechanism to mine high-order feature correlations and achieve global feature fusion enhancement. It also proposes a Full-Process Aggregation and Distribution (FullPAD) paradigm to strengthen feature representation and information flow, as well as optimize gradient propagation for improved detection performance. Meanwhile, depthwise separable convolution (DSConv) [29] and the DSC3k2 lightweight module are adopted to significantly reduce parameter count and computational complexity, achieving better detection accuracy with low computational overhead. As an advanced detection model that balances speed and performance, its architecture is illustrated in Figure 8.

**Figure 9:**
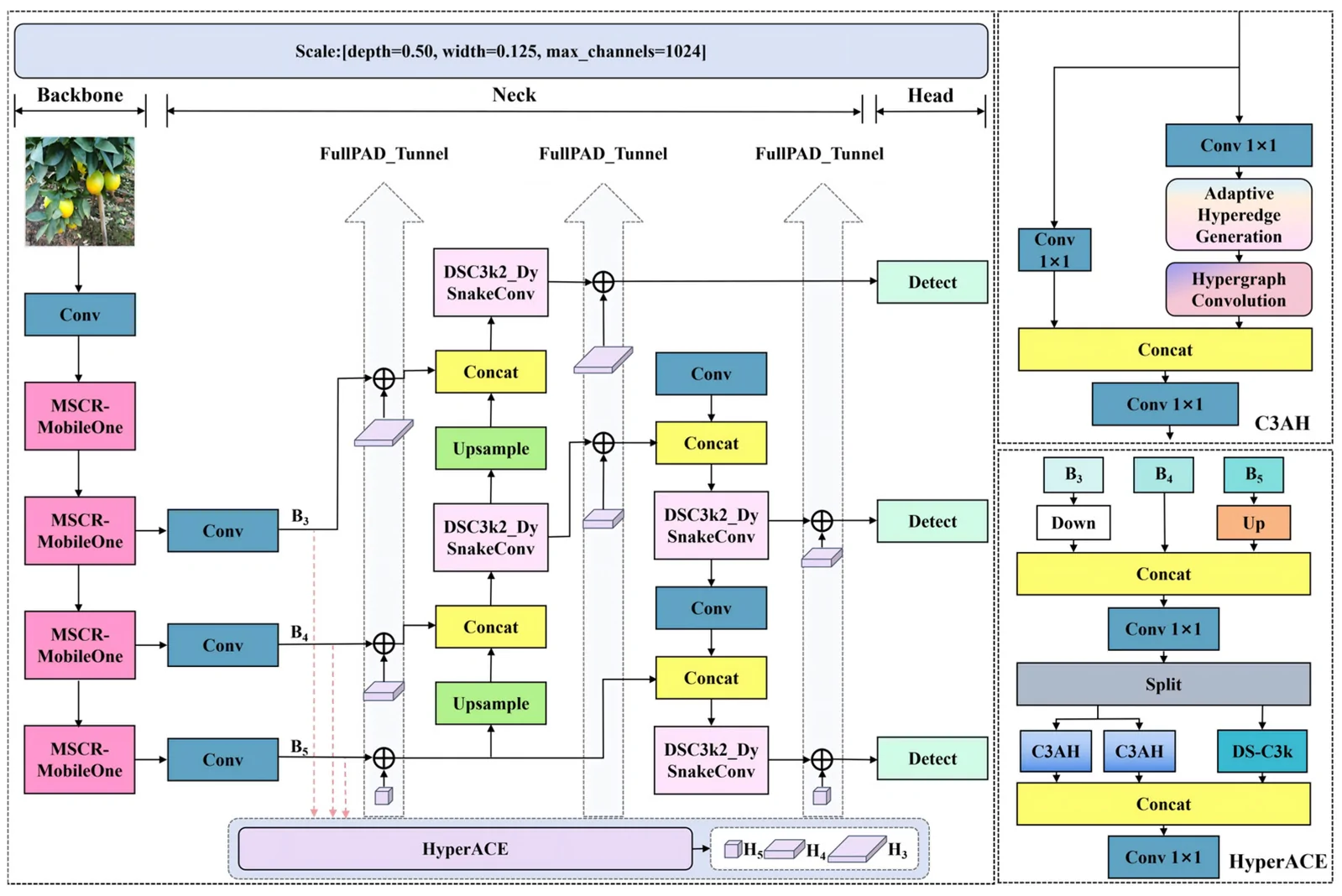
Schematic diagram of the PMDS-YOLO model architecture.

To achieve accurate detection of citrus fruits in complex orchard environments while meeting the requirements of model lightweightness and real-time inference for edge device deployment, this study proposes PMDS-YOLO, a lightweight citrus detection model based on YOLOv13n. This model is optimized in four dimensions: width factor, backbone network, feature extraction module, and post-processing strategy. While significantly reducing model complexity, it improves the detection accuracy of multi-scale and occluded citrus targets. The overall architecture of PMDS-YOLO is shown in Figure 9.

The core improvements include:

1. Width factor compression: The model width scaling factor is compressed to 0.125, proportionally reducing the number of channels in each layer of the network, thereby effectively eliminating channel redundancy and reducing computational cost.
2. Backbone network reconstruction: The backbone network is replaced with a MobileOne structure embedded with the proposed MCSR (MCSR-MobileOne), which reduces the parameter count while enhancing the perception and representation capabilities of multi-scale citrus features.
3. Feature extraction optimization: The DSC3k2 module is improved by adopting DySnakeConv, which further reduces the model’s computational cost while enhancing feature extraction of irregular fruit contours and improving detection robustness against occluded targets.
4. Post-processing strategy optimization: Soft-NMS is introduced to replace the traditional Non-Maximum Suppression (NMS), effectively reducing missed detections of densely overlapping targets and further improving detection accuracy without increasing model parameters.

#### 2.2.1. YOLOv13pico

To meet the deployment requirements of resource-constrained edge devices in smart agriculture scenarios, the YOLO series models achieve flexible scaling of network size through a compound scaling strategy, in which the depth factor regulates the stacking number of basic modules, the width factor uniformly scales the number of convolutional channels in each layer, and the maximum number of channels constrains the upper limit of network feature channels. Although deeper and wider network structures can enhance the visual feature representation capability under complex backgrounds in unstructured orchards, they significantly increase model size and computational overhead, making efficient operation on edge computing platforms difficult [30]. As the lightest native version of the YOLOv13 series, YOLOv13n has a depth factor of 0.5, a width factor of 0.25, and a maximum channel number of 1024. When applied to citrus detection tasks, however, substantial structural redundancy remains. To address this, the width factor was further compressed from 0.25 to 0.125 while keeping the depth factor and maximum channel number unchanged, thereby uniformly halving the number of channels in each layer of the network and constructing an ultra-lightweight YOLOv13pico. The selection of the width factor of 0.125 is based on a trade-off between model lightweightness and feature representation capability. After compression, the number of channels in the deepest layer decreases from 256 to 128, which is still sufficient to maintain discriminative feature representations for citrus targets, and the parameter count drops from 2.46 M to 0.81 M. Further compression to 0.0625, with merely 64 channels in deep layers, would lead to an excessive reduction in feature dimensionality and degrade the target discrimination capability under complex backgrounds. Therefore, the width factor of 0.125 achieves a reasonable balance between lightweight design and feature representation performance.

#### 2.2.2. MCSR

MobileOne achieves significant model compression through structural re-parameterization, but it has inherent limitations in the pursuit of extreme lightweightness. The re-parameterized single-path 3 × 3 convolution has a limited receptive field, making it difficult to effectively capture contextual information of citrus fruits at different scales. Meanwhile, its built-in Squeeze-and-Excitation (SE) attention mechanism only performs simple channel compression and lacks spatial discriminative modeling capability, making it difficult to accurately focus on key fruit regions in complex orchard backgrounds [31]. To address these issues, MCSR is proposed to enhance the multi-scale feature extraction capability and spatial discriminative ability of the MobileOne backbone network. The MCSR module adopts a design of parallel multi-scale extraction and serial attention fusion, preserving the lightweight advantages of MobileOne while compensating for its shortcomings in receptive field and attention mechanisms.

**Figure 10:**
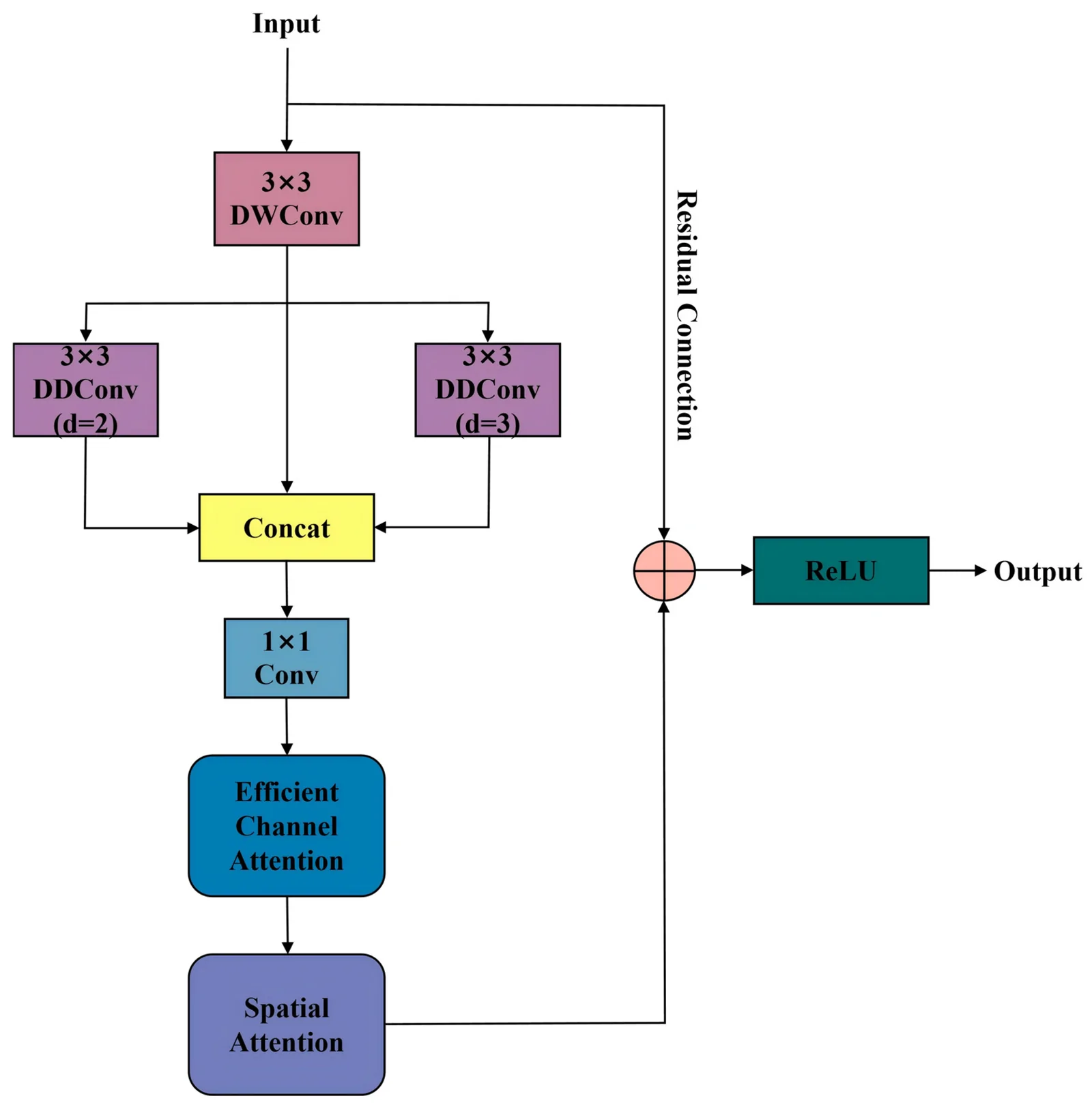
Structure of the MCSR module.

As shown in Figure 10, the input features first pass through a 3 × 3 depthwise convolution (DWConv) [32] to extract local fine-grained features, and a channel compression strategy is adopted to reduce the dimension to one-eighth of the original channels, thereby controlling computational cost.

Subsequently, the compressed features are processed by 3 × 3 depthwise dilated convolutions (DDConv) [33] with dilation rates of 2 and 3, respectively, expanding the equivalent receptive fields to 5 × 5 and 7 × 7, enabling the extraction of multi-scale contextual features. The outputs of the three branches are concatenated along the channel dimension and then restored to the original channel dimension through a 1 × 1 convolution. In the attention weighting stage, MCSR adopts a “channel-space” serial attention mechanism. First, global average pooling compresses the spatial dimension to 1 × 1, followed by an ECA module [34] to generate channel weights, which are then normalized by Sigmoid and multiplied channel-wise with the original features, achieving channel-adaptive weighting. Subsequently, SA [35] applies a 1 × 1 convolution to compress the channel-weighted features to a single channel, generates spatial weights via Sigmoid activation, and performs element-wise multiplication with the feature map to further enhance fruit region responses and suppress background interference. Finally, the MCSR module adds the attention-weighted features to the original input features through a residual connection [36] and outputs them via a ReLU activation function [37]. This design not only preserves original feature information and prevents gradient vanishing but also ensures training stability. The MCSR module as a whole adopts depthwise separable convolutions and channel compression strategies, resulting in extremely low parameter and computational overhead. It effectively enhances the model’s multi-scale feature extraction capability for citrus targets in complex orchard scenes with almost no increase in inference cost.

#### 2.2.3. MCSR-MobileOne

**Figure 11:**
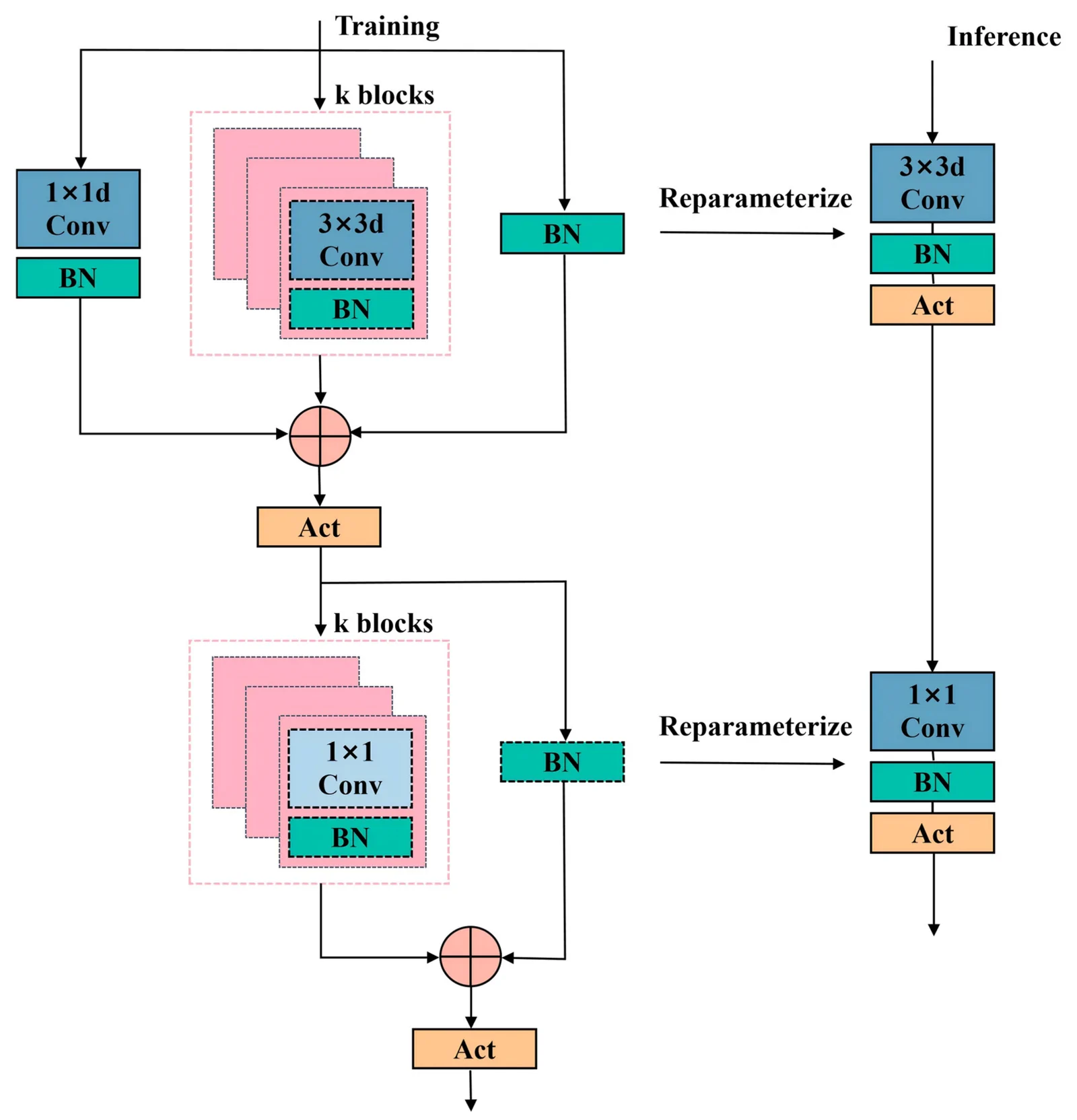
Architecture of the MobileOne block.

In natural orchard environments, citrus targets exhibit a wide span of scale distribution, complex background interference, and limited computational power and storage resources on edge devices. The native YOLOv13n backbone network exhibits substantial computational redundancy and insufficient capability in feature representation and contextual modeling for multi-scale citrus targets, making it difficult to balance detection accuracy and edge deployment requirements. As shown in Figure 11, MobileOne [38], based on the concept of structural re-parameterization, constructs a heterogeneous topology of “multi-branch for training, single-branch for inference” and uses depthwise separable convolution as the basic unit. It can significantly reduce parameter count and computational cost while maintaining basic feature extraction capability, making it a preferred structure for lightweight backbone networks. However, this network still has inherent shortcomings when applied to complex orchard detection tasks. After re-parameterization fusion, only a single 3 × 3 convolution path remains, resulting in a fixed and limited effective receptive field, which makes it difficult to fully capture cross-scale contextual dependencies of citrus fruits. Meanwhile, its built-in SE attention mechanism is limited to channel-wise feature recalibration and lacks spatial location awareness and long-range dependency modeling capability, making it difficult to adaptively filter and focus on citrus target regions under complex backgrounds with overlapping branches and uneven illumination.

To address these shortcomings of MobileOne, including limited receptive field, weak multi-scale modeling capability, and lack of spatial attention, the proposed MCSR is embedded into the native MobileOne feature extraction pipeline. The constructed MCSR-MobileOne effectively compensates for the insufficient multi-scale representation capability of the original network while inheriting the extreme lightweight advantages of MobileOne and maintaining low parameter count and low computational cost. On the one hand, it broadens the dynamic receptive field range, enabling refined feature modeling of citrus fruits of varying sizes. On the other hand, it enhances the joint channel-space feature filtering capability. This significantly improves the model’s robustness and discriminability in extracting citrus target features under complex orchard interference backgrounds.

#### 2.2.4. DySnakeConv

**Figure 12:**
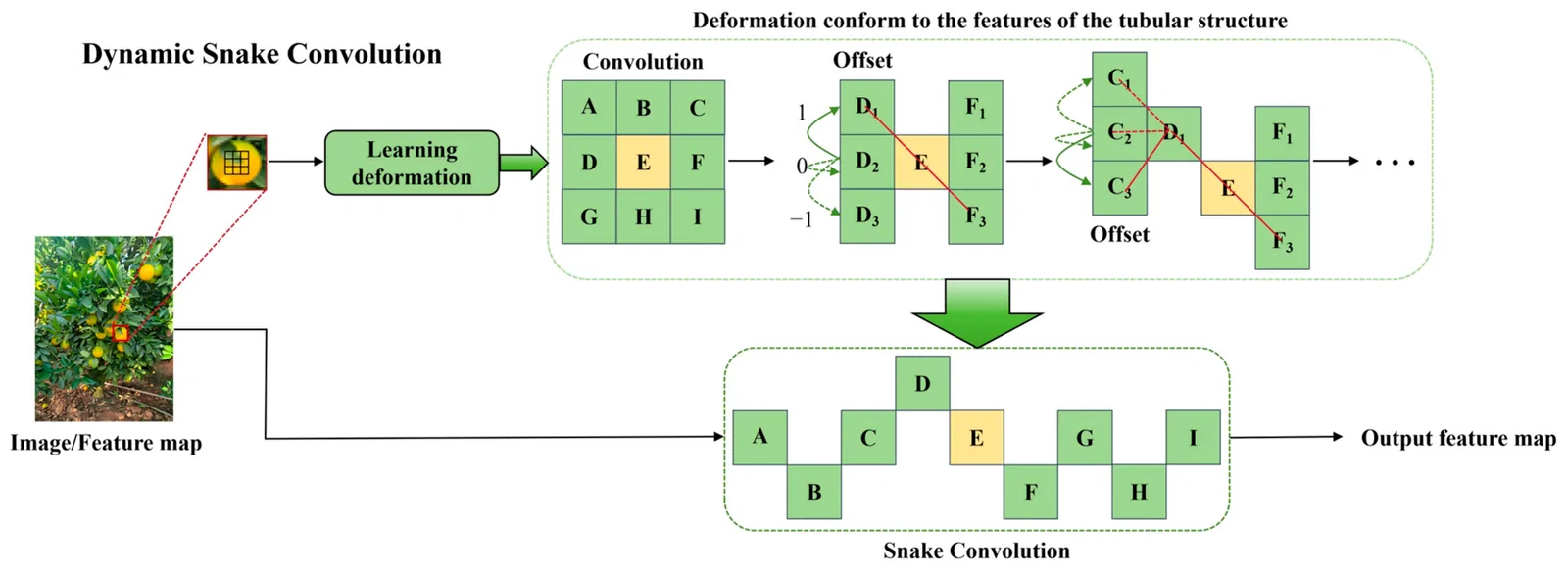
Architecture of the DySnakeConv module.

To address the issues of irregular citrus contours and severe occlusion interference in orchard scenarios, the DSC3k2 module in the original YOLOv13n adopts static regular convolution kernels, which struggle to adaptively fit the curved contours of fruits, resulting in insufficient feature extraction and computational redundancy. To this end, the depthwise separable bottleneck structure (DSBottleneck) inside the original DSC3k2 module is replaced with a DSC3k unit integrated with DySnakeConv [39], while preserving the lightweight architecture of the original module. As shown in Figure 12, DySnakeConv adopts a three-branch parallel architecture. The standard convolution branch serves as the basic feature extraction unit to preserve the general structural information of the target. The two dynamic snake convolution branches along the x-axis and y-axis adaptively learn the offsets of sampling points through lightweight offset convolution. After being constrained to the [−1, 1] interval by the Tanh function, an iterative accumulation strategy is used to sequentially determine the positions of each sampling point, and bilinear interpolation is employed to achieve feature sampling under continuous coordinates, enabling the strip-shaped convolution kernel to adaptively extend and conform to the curved contours of the target, thereby preventing the receptive field from drifting outside the target. This mechanism breaks the fixed constraints of regular convolution kernels through snake-like sampling, adaptively fitting the irregular contours and occlusion boundaries of citrus fruits while maintaining a lightweight structure, thereby enhancing the extraction capability of edge and local features.

#### 2.2.5. Soft-NMS

In the object detection pipeline, models typically generate multiple candidate bounding boxes with high overlap for the same target. NMS [40] adopts a hard thresholding strategy: when the Intersection over Union (IoU) between a candidate box and the highest-confidence prediction box exceeds a preset threshold, its confidence is directly set to zero, and the box is discarded. This method works well in sparse target scenarios. However, in complex environments with dense targets such as citrus orchards, it tends to misclassify true positive instances as redundant detections due to highly overlapping bounding boxes of different targets, leading to an increased missed detection rate. To alleviate this issue, the Soft-NMS [41] mechanism is introduced to replace the original NMS module. Sof-NMS exhibits the same asymptotic computational complexity of O(N^2^) as standard NMS, where N denotes the number of candidate bounding boxes. Their core difference lies in the confidence-update strategy: Soft-NMS employs a Gaussian decay function, whereas conventional NMS performs hard-threshold filtering. Both algorithms require sorting and pairwise Intersection-over-Union (IoU) calculations. Soft-NMS only introduces elementary arithmetic operations for Gaussian decay on overlapping candidate boxes without additional sorting or iterative-search procedures, yielding negligible computational overhead. Instead of directly removing candidate boxes with high IoU, Soft-NMS penalizes them through a confidence decay function, thereby reducing the miss detection rate. The Gaussian decay function of Soft-NMS is formulated as follows:

(1) $$s_{i}=s_{i}e^{-\frac{\mathrm{iou}{\left(M,b_{i}\right)}^{2}}{\sigma }},\forall b_{i}\notin D$$

In the formula, σ represents the decay factor, which controls the penalty intensity. Based on the accuracy requirements of the grading task, σ is set to 0.2, and the confidence threshold is set to 0.45 in this study, thereby improving precision while effectively controlling missed detections.

### 2.3. Experimental Setup

All models in this study were trained and validated under a unified hardware platform and software environment. The hardware platform consisted of an NVIDIA GeForce RTX 4060 graphics card, an AMD Ryzen 9 7940HX processor, and 16 GB of memory, ensuring consistent computational resources. The software environment was built on Python 3.9, utilizing the PyTorch 2.5.1 deep learning framework and the CUDA 12.7 parallel computing library, with PyCharm 2024.1 Professional Edition as the integrated development environment, ensuring stable and compatible model operation. To ensure experimental rigor, comparative models with identical architectures employed exactly the same hyperparameters, while models with different architectures (Faster R-CNN, SSD, RetinaNet, and YOLO-series variants) used their respective hyperparameter configurations. Given the inherent differences in network structure, optimization strategies, and training paradigms across these architectures, hyperparameters were individually tuned for each model to achieve optimal performance on the citrus detection task. The main training configurations of the compared models are presented in Table 1.

### 2.4. Evaluation Metrics

To evaluate model performance, Precision (P), Recall (R), and mean Average Precision (mAP) were adopted as the core detection metrics. The calculation formulas are as follows:

(2) $$P=\frac{TP}{TP+FP}\times 100\%$$

(3) $$R=\frac{TP}{TP+FN}\times 100\%$$

(4) $$mAP=\frac{\sum_{k=1}^{K}AP(k)}{K}\times 100\%$$

(5) $$AP=\int_{0}^{1}P(R)dR$$

In the formulas, TP represents the number of correctly detected targets in the test set, FP represents the number of falsely detected targets, and FN represents the number of missed targets. AP (Average Precision) is the area under the precision-recall curve, used to characterize the detection accuracy of a single class, while mAP (mean Average Precision) is the average of AP values across all detected classes, which can comprehensively reflect the overall detection performance of the model.

The parameter count (Parameters), computational cost (FLOPs), inference frame rate (FPS), and model size were selected to evaluate the lightweight level and real-time performance of the models. FPS was calculated on a GPU device by performing 200 warm-up iterations to eliminate the impact of cold starts, and the average inference time of 1000 single-frame inferences was used as the per-frame inference time. The calculation formulas are as follows:

(6) $$\mathrm{Parameters}=\sum \left(K\times K\times C_{\mathrm{in}}\times C_{\mathrm{out}}\right)$$

(7) $$\mathrm{FLOPs}=\sum \left(H\times W\times K\times K\times C_{\mathrm{in}}\times C_{\mathrm{out}}\right)$$

(8) $$FPS=\frac{1}{\mathrm{Inference}\;\mathrm{Time}\;(s)}$$

In the formula, H × W represents the height and width of the output feature map, K represents the convolution kernel size, Cin represents the number of input channels, Cout represents the number of output channels, and Inference Time (s) represents the single-frame inference time.

To evaluate the estimation accuracy of PMDS-YOLO for citrus diameter, data fitting was performed using the estimated diameter values and actual measured values of 100 citrus fruits as samples. The coefficient of determination (R^2^) was adopted to measure the goodness of fit between predicted and measured values; the root mean square error (RMSE) was used to quantify the magnitude of estimation error, and the mean relative error (MRE) and mean absolute error (MAE) were employed to assess the systematic deviation between the initially estimated diameter and the measured diameter. The calculation formulas are as follows:

(9) $$R^{2}=1-\frac{\sum_{i=1}^{n}{\left(y_{i}-{\hat{y}}_{i}\right)}^{2}}{\sum_{i=1}^{n}{\left(y_{i}-\bar{y}\right)}^{2}}$$

(10) $$RMSE=\sqrt{\frac{1}{n}\sum_{i=1}^{n}{\left(y_{i}-{\hat{y}}_{i}\right)}^{2}}$$

(11) $$\mathrm{MAE}=\frac{1}{n}\sum_{i=1}^{n}|y_{i}-{\hat{y}}_{i}|$$

(12) $$\mathrm{MAPE}=\frac{1}{n}\sum_{i=1}^{n}\left|\frac{y_{i}-{\hat{y}}_{i}}{y_{i}}\right|\times 100\%$$

In the formulas, $y_{i}$ represents the measured true diameter, ${\hat{y}}_{i}$ represents the diameter predicted by the model, $\bar{y}$ represents the mean of the measured diameters, and *n* represents the number of samples.

## 3. Results

### 3.1. Comparative Experiment of Different Object Detection Models

To verify the comprehensive performance advantages of the PMDS-YOLO model in citrus detection tasks, comparative experiments were conducted under identical experimental settings with Faster R-CNN, SSD, RetinaNet, and various lightweight YOLO series models.

As shown in Table 2, although Faster R-CNN, SSD, and RetinaNet achieved certain detection accuracy, their parameter counts were excessively large, making it difficult to meet the deployment requirements of edge devices. In contrast, the YOLO series achieved a better balance between accuracy and model size due to their streamlined network structures and efficient feature fusion mechanisms. Among them, YOLOv13n exhibited the best balance between accuracy and lightweightness, achieving an mAP@0.5:0.95 of 85.42% with a model size of only 5.20 MB, and was therefore selected as the baseline model for this study. The proposed PMDS-YOLO model significantly reduced the parameter count while substantially improving detection accuracy. It achieved a precision of 97.43% with a model size of only 1.44 MB. Under comparable detection accuracy to YOLOv11n and YOLOv13n, PMDS-YOLO achieves a 76.4% parameter reduction, offering stronger deployment advantages for real-time citrus detection tasks on edge devices.

### 3.2. Ablation Experiments

To quantitatively analyze the individual contributions and synergistic gains of each improvement strategy and to verify the effectiveness of the proposed algorithmic framework, eight ablation experiments were conducted using the native YOLOv13n as the baseline model, focusing on three core improvements: network scaling (Pico), MCSR-MobileOne backbone replacement, and DSC3k2 module optimization. All experimental groups were trained and tested under identical hardware environments, dataset configurations, and hyperparameter settings to ensure strict consistency of experimental variables. The evaluation metrics selected were mAP@0.5:0.95, Precision (P), parameter count, and FLOPs, which comprehensively assess the detection accuracy and lightweight deployment capability of the models. The experimental results for each group are presented in Table 3. The baseline YOLOv13n model achieved a mAP@0.5:0.95 of 85.42%, a precision of 95.35%, 2.46 M parameters, and 6.4 G FLOPs.

The single-module ablation results reveal the individual effects of each improvement strategy. When width factor compression (Pico) was introduced alone, the parameter count was reduced by 67.07%, yet mAP@0.5:0.95 declined by 0.9 percentage points. This result indicates that the primary role of Pico is model compression rather than accuracy enhancement, achieving substantial lightweightness at the cost of controlled accuracy loss. In contrast, the MCSR-MobileOne backbone alone reduced the parameter count by 28.86% while simultaneously improving mAP@0.5:0.95 by 0.38 percentage points, confirming its efficacy in multi-scale feature extraction and background discrimination. Similarly, DySnakeConv alone reduced the parameter count by 6.91% while increasing mAP@0.5:0.95 by 0.78 percentage points, validating its enhanced adaptability to irregular fruit contours.

Multi-module combination ablations further revealed the synergistic characteristics of the improvement components. Without the scaling strategy, the integration of MCSR-MobileOne and DySnakeConv (Model 6) established an effective feature complementarity mechanism, reducing the parameter count by 39.84% and improving mAP@0.5:0.95 by 0.11 percentage points over the baseline, thereby demonstrating the positive gain of dual-module integration. Under width compression, the combination of MCSR-MobileOne and DySnakeConv (Model 7) achieved comprehensive network redundancy reduction, lowering the parameter count to 0.58 M and FLOPs to 2.3 G; however, the mAP@0.5:0.95 reached only 84.77%, remaining 0.65 percentage points below the baseline. This observation suggests that the enhancements introduced by the backbone and feature extraction modules were partially offset following width compression.

Notably, with the subsequent incorporation of Soft-NMS (Model 8), model performance was further elevated: mAP@0.5:0.95 reached 86.41%, representing gains of 1.64 percentage points over Model 7 and 0.99 percentage points over the baseline; precision reached 97.43%, an improvement of 1.50 percentage points over Model 7 and 2.08 percentage points over the baseline; and parameter count and computational cost remained unchanged. This result demonstrates that Soft-NMS effectively mitigates missed detections of densely overlapping targets, further improving detection accuracy without incurring additional parameter overhead.

**Figure 13:**
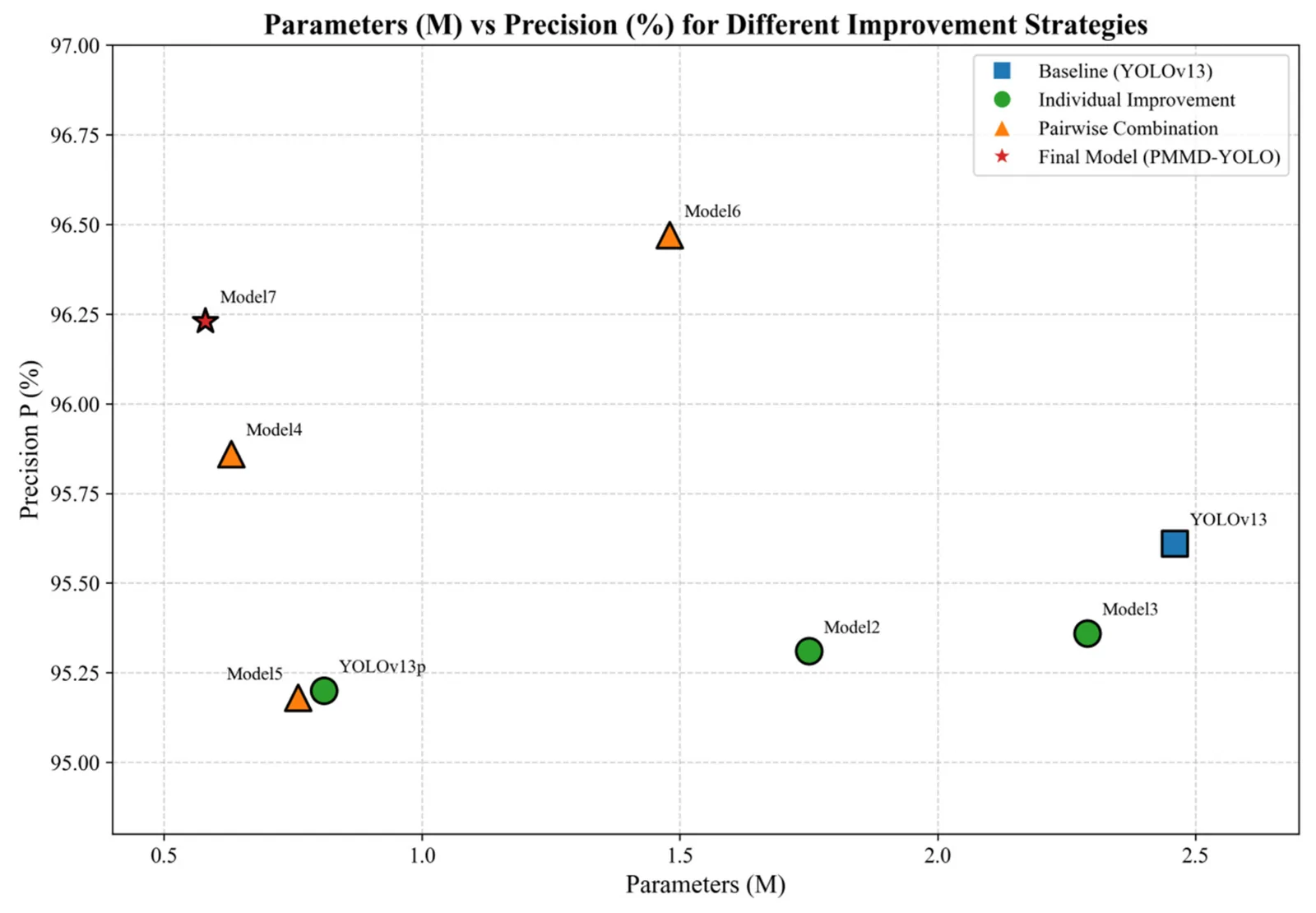
Precision-parameter trade-off analysis.

These results demonstrate a clear division of labor and synergistic complementarity among the modules in the complete combination: Pico is responsible for model compression and lightweightness, establishing the parameter foundation for edge deployment; MCSR-MobileOne and DySnakeConv enhance representational capacity at the backbone and feature extraction levels; and Soft-NMS further mitigates missed detections in dense scenarios without incurring additional parameter overhead. The complete model (Model 8) achieves a 76.4% reduction in parameter count and a 64.1% reduction in computational cost, while improving mAP@0.5:0.95 by 0.99 percentage points and precision by 2.08 percentage points, realizing an effective integration of lightweightness and high accuracy. The precision-parameter trade-off analysis of the ablation experiments is shown in Figure 13.

### 3.3. Validation of MCSR Attention Effectiveness

To validate the effectiveness of the proposed MCSR, a model integrating width factor compression, replacement of the backbone network with the original MobileOne, and optimization of the DSC3k2 structure with DySnakeConv was used as the baseline. Comparative experiments were conducted by embedding four mainstream attention mechanisms (CA, CBAM, EMA, and GAM) and the proposed MCSR attention mechanism into the MobileOne network, respectively. All models were trained and tested under identical experimental environments and hyperparameter configurations.

**Figure 14:**
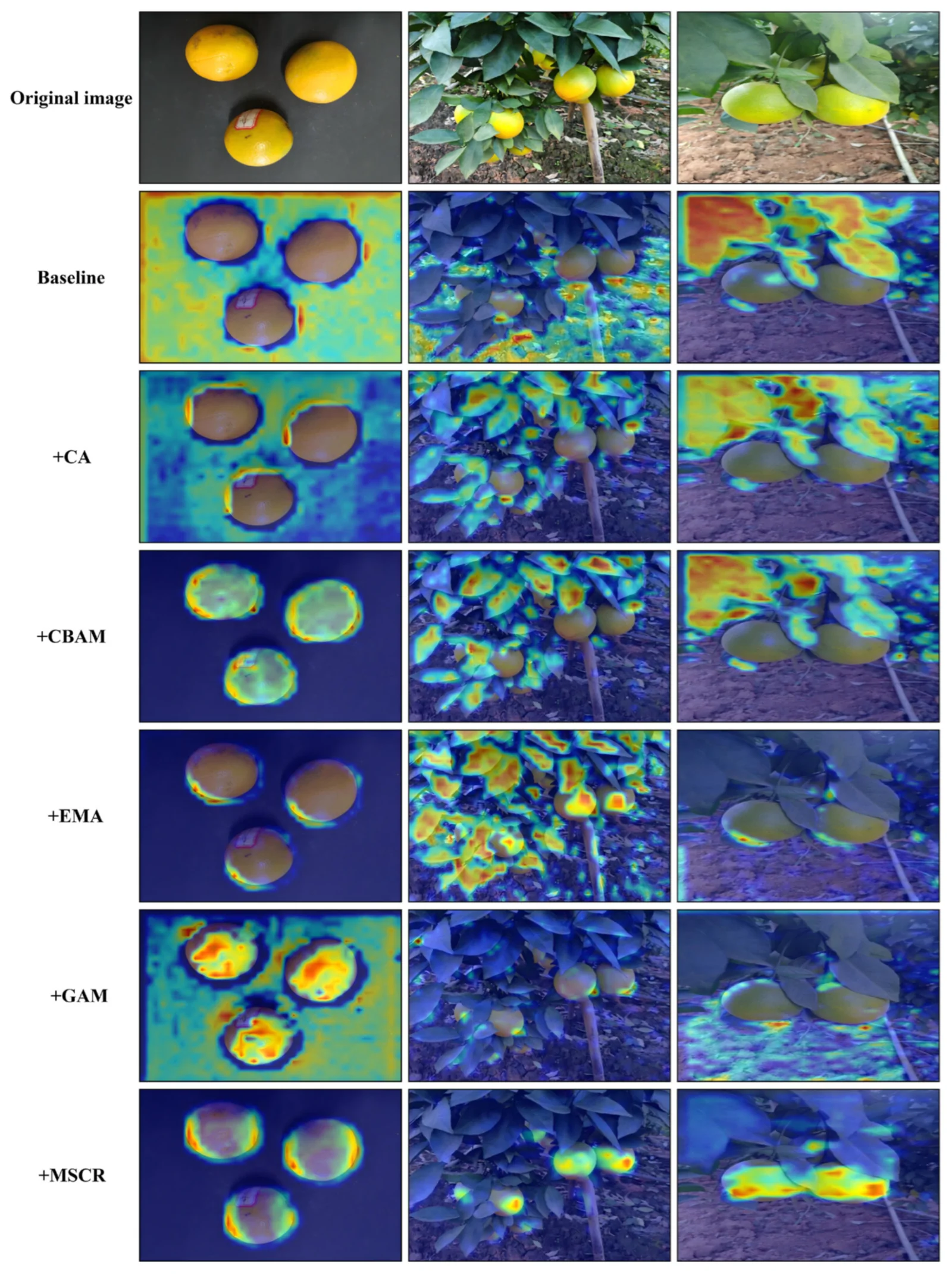
EigenCAM visualization of different attention mechanisms. The color represents the activation intensity of the model’s high-level semantic features, ranging from low (blue) to high (red).

The experimental results are presented in Table 4. The baseline model (PMD) achieved an mAP@0.5:0.95 of 83.60% and a precision of 94.14%. After embedding MCSR, the mAP reached 84.77% (a gain of 1.17 percentage points) and precision reached 95.93% (a gain of 1.79 percentage points). Among the four attention mechanisms, CBAM performed similarly to the baseline (83.56%), while CA, EMA, and GAM achieved smaller improvements than MCSR. The core of MCSR lies in the architectural fusion of multi-scale context extraction and serial channel–spatial attention. The module employs parallel multi-branch depthwise dilated convolutions (dilation rates of 2 and 3) to construct a multi-scale receptive field, combined with a channel-first, spatial-second serial attention refinement structure, to meet the feature extraction demands of complex orchard scenarios. Unlike SE and ECA, which rely solely on global pooling, this structure integrates multi-scale contextual information across 5 × 5 and 7 × 7 receptive fields. Meanwhile, compared with the parallel stacking design of CBAM, the serial optimization architecture helps alleviate the entanglement of features across different attention branches. Furthermore, by incorporating depthwise separable convolutions and channel compression strategies, the module introduces only 0.01 M additional parameters, enabling lightweight integration into the MobileOne backbone network. To intuitively compare the target focusing and background suppression capabilities of different attention mechanisms, EigenCAM was employed to visualize the high-level semantic features of the third layer of the backbone network. The results, shown in Figure 14, indicate that the baseline model exhibited relatively dispersed feature responses susceptible to background noise interference. After embedding CA, CBAM, EMA, and GAM, the models focused on citrus targets to some extent, but the response regions still contained background information. In contrast, MCSR concentrated high-response regions precisely on the main bodies and edge contours of the fruits, with effective suppression of background responses. The quantitative and visualization results demonstrate that MCSR, with only 0.01 M additional parameters, achieves optimal detection accuracy and target focusing, fully verifying its superiority in lightweight design and feature enhancement.

### 3.4. Comparative Experiment on Feature Extraction

To identify the optimal DSC3k2 improvement scheme compatible with YOLOv13n, this section employs YOLOv13n as the baseline. The original DSC3k2 module is improved by adopting DySnakeConv, BiFormer, GnConv, iRMB, and RFAConv, respectively, and comparative experiments are conducted to quantitatively evaluate the effectiveness of each strategy from the two dimensions of detection accuracy and parameter count.

**Figure 15:**
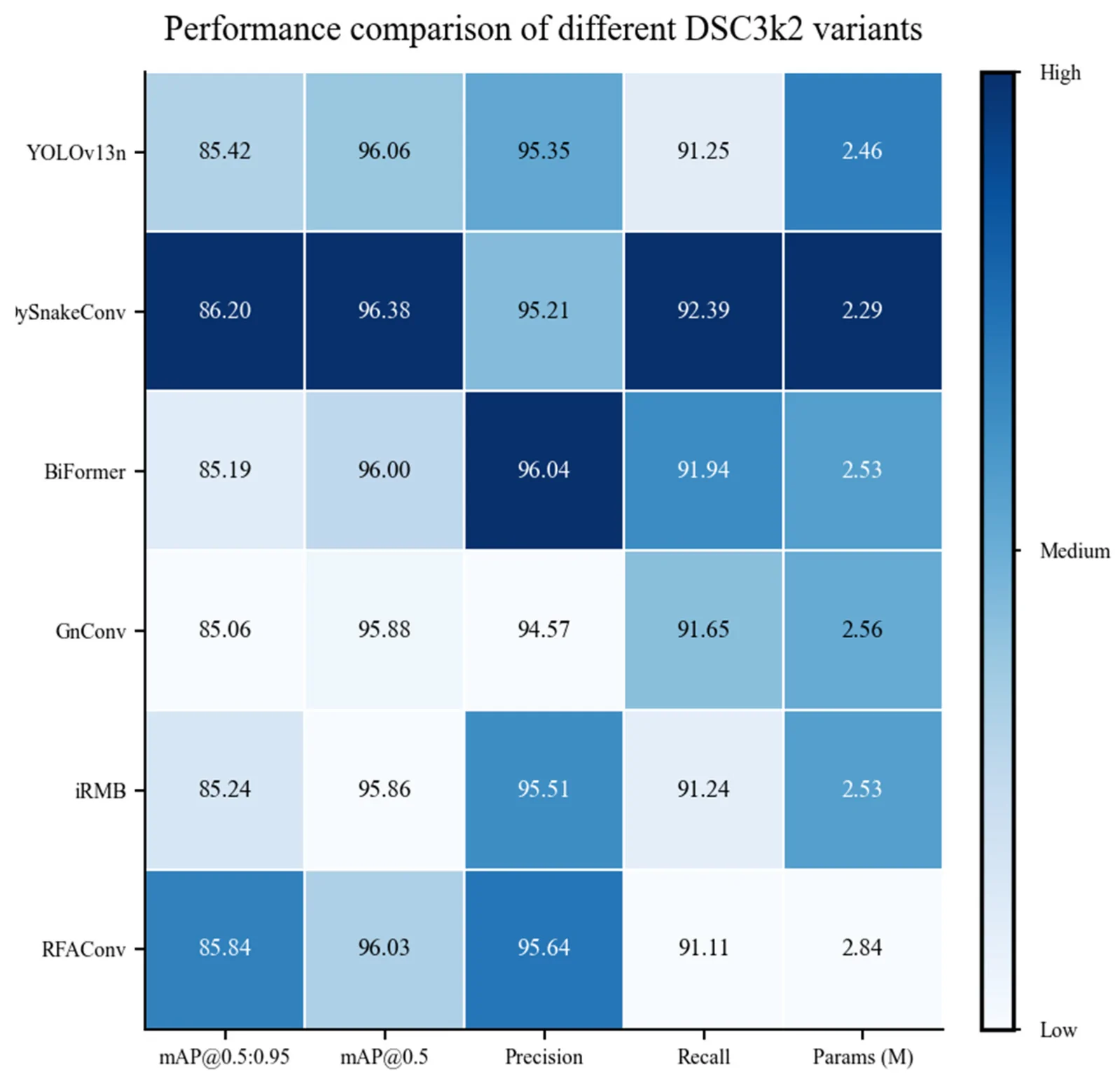
Performance comparison of different DSC3k2 variants.

As shown in Table 5 and Figure 15, most of the improved strategies enhanced the detection performance of the model to varying degrees. Among them, RFAConv achieved an mAP@0.5:0.95 of 85.84%, an improvement of 0.42 percentage points over the baseline, but its parameter count increased to 2.84 M (an increase of 15.45%), introducing significant computational redundancy. BiFormer achieved the highest precision among all compared models at 96.04%, but its parameter count was 2.53 M, which is higher than that of the baseline. GnConv and iRMB achieved limited accuracy improvements, and their parameter counts were also higher than that of the baseline. Considering both accuracy improvement and lightweight requirements, the DySnakeConv improvement strategy increased mAP@0.5:0.95 by 0.78 percentage points (from 85.42% to 86.20%) while increasing the parameter count by only 0.17 M (from 2.46 M to 2.29 M), effectively controlling model size while improving detection accuracy and achieving a favorable balance between accuracy and lightweight performance. Furthermore, DySnakeConv achieved a recall of 92.39%, which is also superior to the other compared models. These results indicate that DySnakeConv can effectively control model size while improving detection accuracy, making it more suitable for deployment scenarios requiring low computational power and high real-time performance on edge computing platforms.

### 3.5. Comprehensive Performance Analysis of PMDS-YOLO

To comprehensively evaluate the overall performance of the proposed model, quantitative comparisons between PMDS-YOLO and the baseline model YOLOv13n were conducted from three dimensions: detection accuracy, lightweightness, and inference speed.

**Figure 16:**
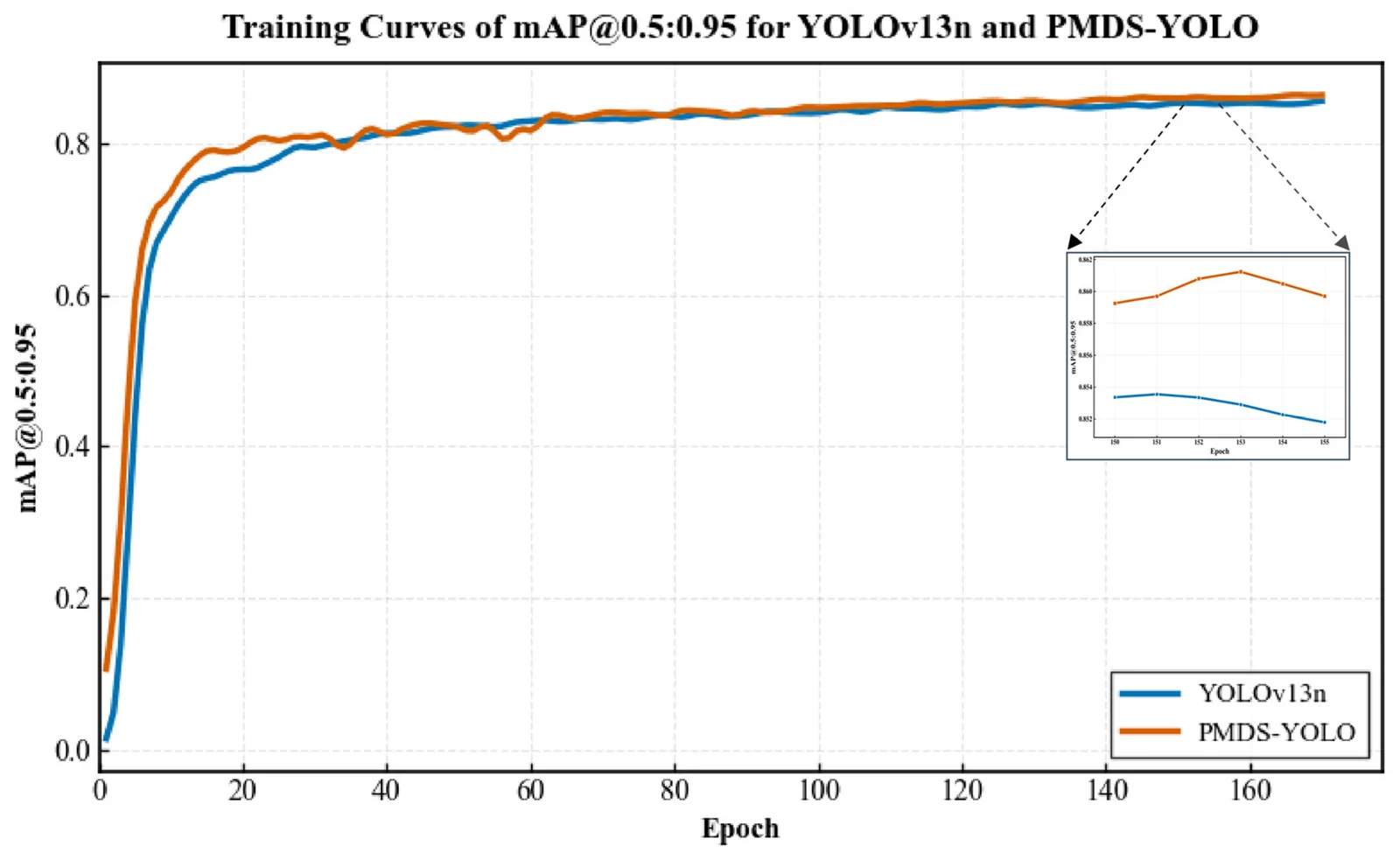
Training Curves of mAP@0.5:0.95 for YOLOv13n and PMDS-YOLO.

To assess statistical reliability, both YOLOv13n and PMDS-YOLO were trained with four different random seeds (0, 1, 2, and 42), and the results are reported as mean ± standard deviation. As shown in Table 6 and Figure 16, PMDS-YOLO achieved a mean mAP@0.5:0.95 of 86.22% ± 0.24% and a mean precision of 97.03% ± 0.42%, representing improvements of 0.97 and 1.93 percentage points, respectively, over the baseline model (85.25% ± 0.21% and 95.10% ± 0.19%). The parameter count and model size were reduced to 0.58 M and 1.44 MB, respectively, corresponding to reductions of 76.4% and 72.3% compared to the baseline (2.46 M and 5.20 MB). The inference frame rate reached 48.0 FPS, an increase of 66.7% over the baseline (28.8 FPS). These results demonstrate that PMDS-YOLO maintains consistent performance advantages across different random seeds, confirming the statistical reliability of the proposed improvements.

**Figure 17:**
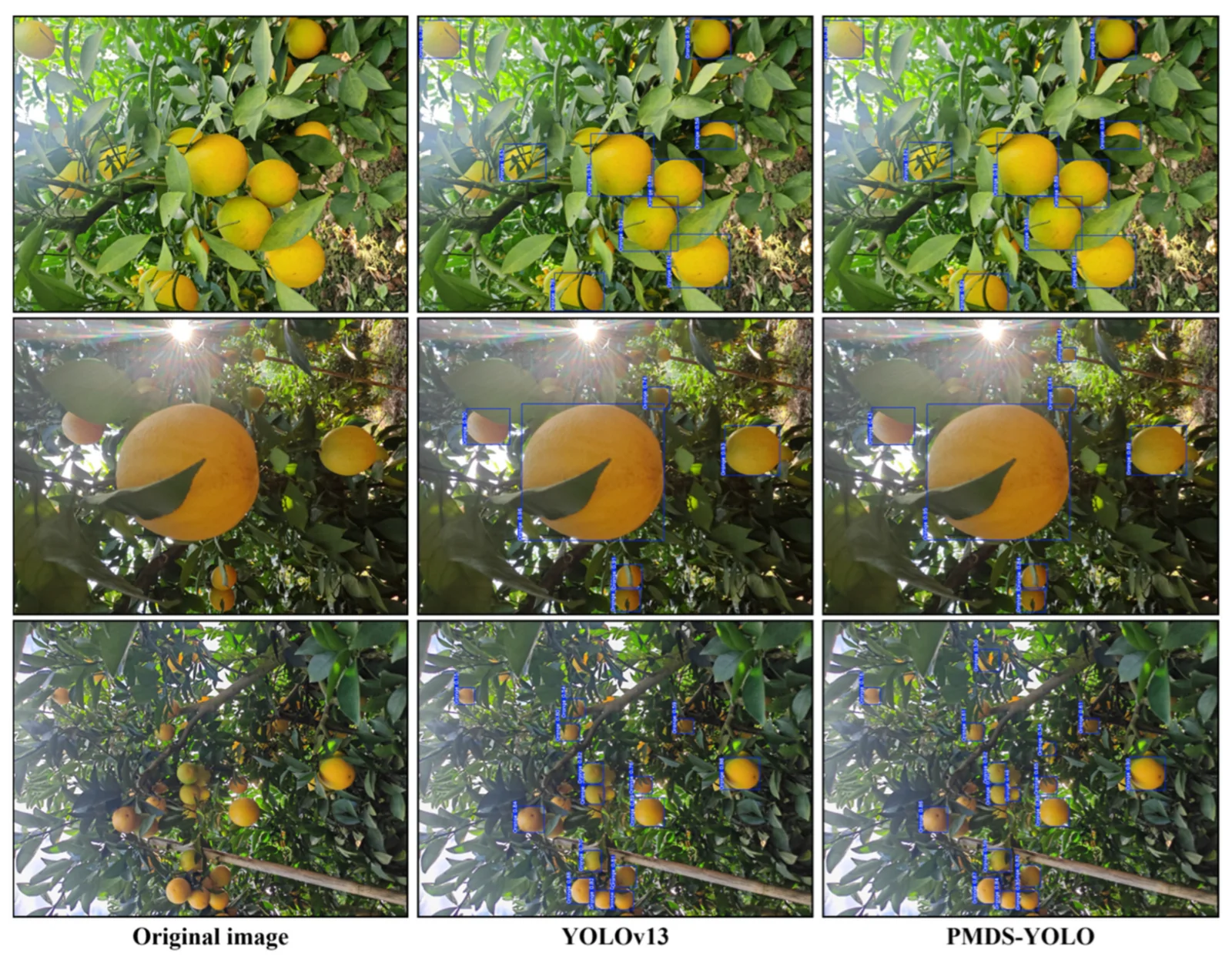
Detection result comparison between PMDS-YOLO and the baseline model.

As illustrated in Figure 17, compared with the baseline model, PMDS-YOLO exhibited fewer missed detections and false detections in densely overlapping and occluded scenarios, with higher detection confidence. In summary, PMDS-YOLO achieves an optimal balance among accuracy, lightweightness, and real-time performance, making it more suitable for real-time citrus detection and diameter estimation tasks on edge devices.

### 3.6. Deviation Analysis and Regression Calibration for Diameter Estimation

To achieve non-contact, low-cost, and accurate estimation of citrus fruit diameter, this study proposes a two-step calculation strategy of “object detection-scale reference-regression calibration”. The proposed PMDS-YOLO model is used to simultaneously detect citrus fruits and a 25 mm standard coin in the same image, and the bounding box heights of both are extracted. The initial citrus fruit diameter is estimated according to Equation (13):

(13) $$D_{est}=\frac{h_{fruit}}{h_{coin}}\times D_{coin}$$

where h_fruit_ is the bounding box height of the citrus fruit, h_coin_ is the bounding box height of the coin, and D_coin_ = 25.0 mm.

This method estimates fruit diameter based on similar triangles, using the ratio of bounding box heights between the fruit and a 25 mm coin in the same image. The underlying geometric assumption is that the coin and fruit are approximately coplanar and that the bounding box height direction is parallel to the image plane. However, this assumption is difficult to strictly satisfy in practice: perspective distortion, irregular fruit shapes, occlusion, and oblique viewing angles all cause the bounding box height to deviate from the true diameter. To compensate for these geometric approximations, a quadratic polynomial regression is applied to calibrate the initial estimates.

**Figure 18:**
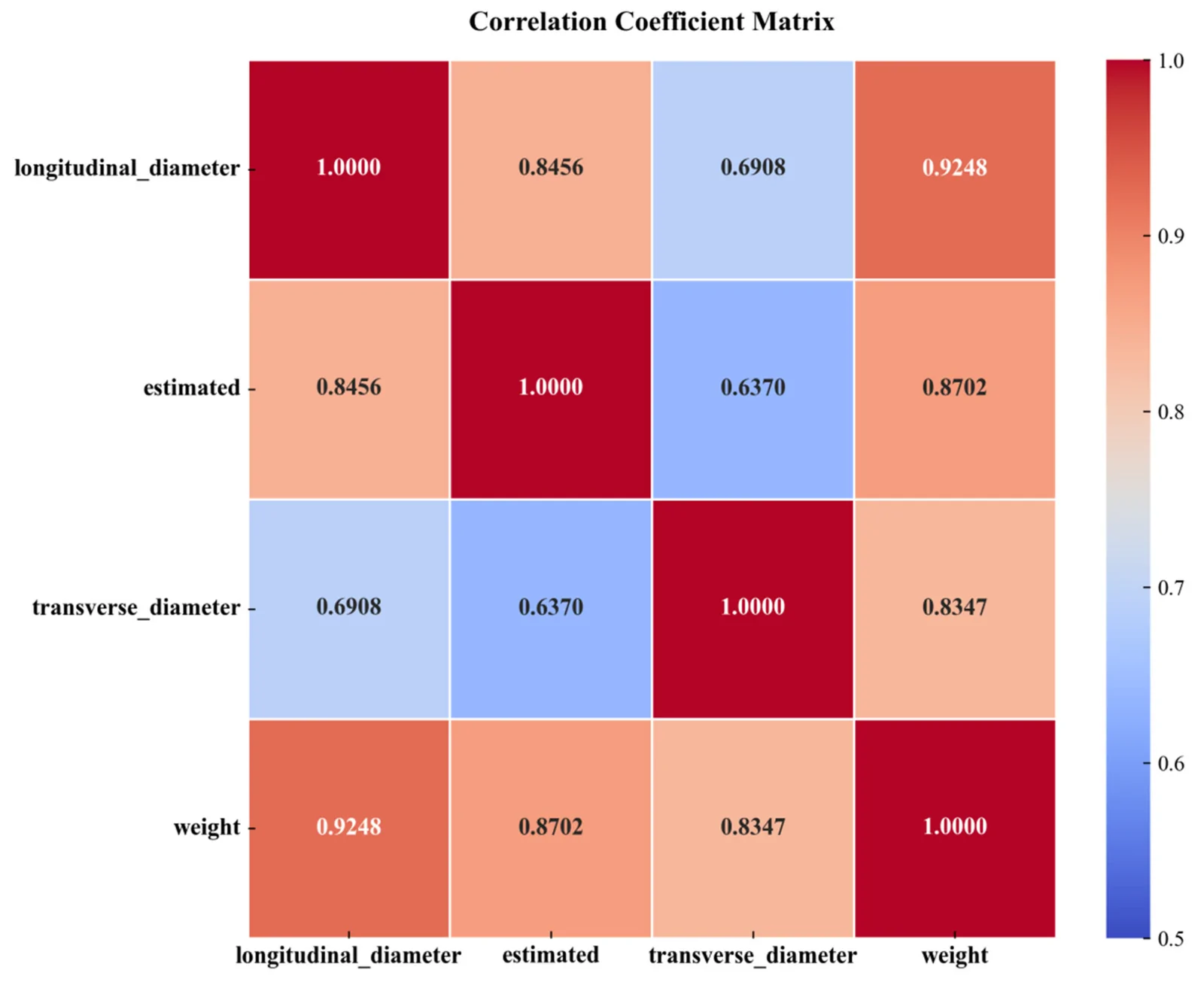
Correlation heatmap of diameter-related variables.

As shown in Figure 18, the initial estimated value exhibited the highest correlation coefficient with single-fruit weight (r = 0.870), and the correlation coefficient with transverse diameter was 0.848, further indicating that the estimated value after reference object conversion effectively retains fruit scale information and possesses a strong linear correlation. Meanwhile, the diameter showed a high positive correlation with single-fruit weight (r = 0.925), validating the rationality of using diameter as a core characterization indicator for citrus size and weight.

**Figure 19:**
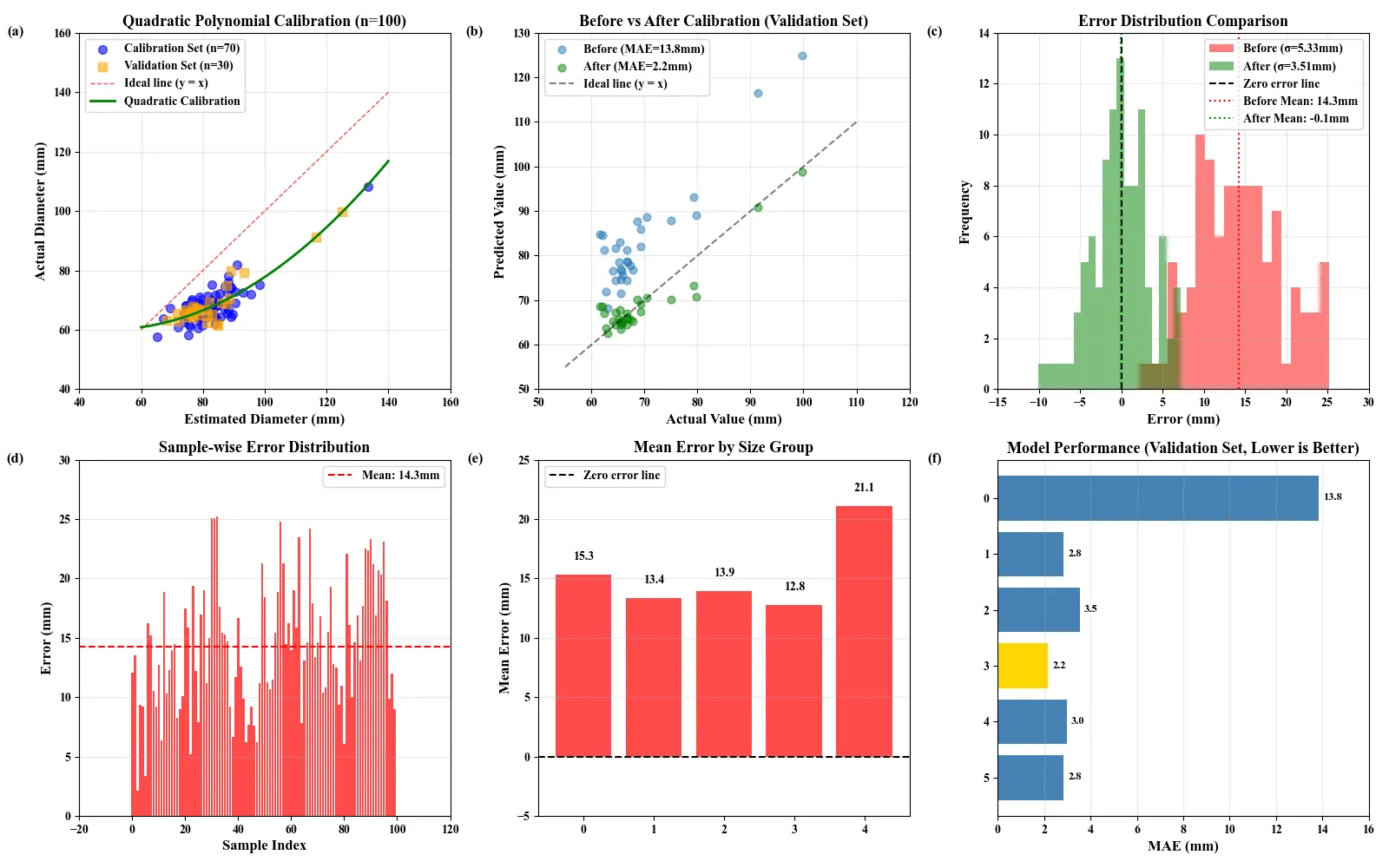
Calibration analysis of citrus diameter estimation: (**a**) Quadratic polynomial regression calibration scatter plot (calibration set *n* = 70, validation set *n* = 30); (**b**) Comparison of estimated values before and after calibration on the validation set; (**c**) Error distribution comparison; (**d**) Sample-wise error distribution; (**e**) Mean error by size group; (**f**) Model performance comparison on the validation set (MAE).

As shown in Figure 19, on the full set of 100 samples, the initial estimated values exhibited a significant systematic deviation from the measured diameters, with a mean absolute error (MAE) of 14.28 mm. Size-grouped error analysis indicated that the initial estimation error increased with fruit size. Notably, for large fruits with a diameter exceeding 80 mm, the average error reached 21.10 mm, showing a clear overestimation trend. These deviations mainly originated from nonlinear errors introduced by factors such as image perspective distortion, the lack of strict coplanarity between the coin and the fruit, and irregular fruit contours. To eliminate the aforementioned nonlinear deviations, the 100 samples were randomly divided into a calibration set (70 samples) and an independent validation set (30 samples) at a ratio of 7:3. Simple linear regression, quadratic polynomial regression, logarithmic regression, and ridge regression were respectively fitted on the calibration set, and the resulting models were applied to the independent validation set to evaluate their predictive performance. The quadratic polynomial regression model takes the initial estimated diameter D_est_ as the independent variable and the measured true diameter D_true_ as the dependent variable. The regression coefficients are estimated by the least-squares method. The model is expressed in Equation (14), where a, b, and c are the parameters to be estimated:

(14) $$D_{true}=a\times D_{est}^{2}+b\times D_{est}+c$$

As shown in Table 7, all four regression models significantly reduced the estimation error, among which quadratic polynomial regression performed the best, reducing the MAE to 2.15 mm, RMSE to 3.16 mm, and achieving an R^2^ of 0.85 on the independent validation set. The optimal calibration formula is:

(15) $$D_{true}=0.006923\times D_{est}^{2}-0.685667\times D_{est}+77.1344$$

where D_true_ is the diameter calibrated by the quadratic polynomial, and D_est_ is the initial estimated diameter.

**Figure 20:**
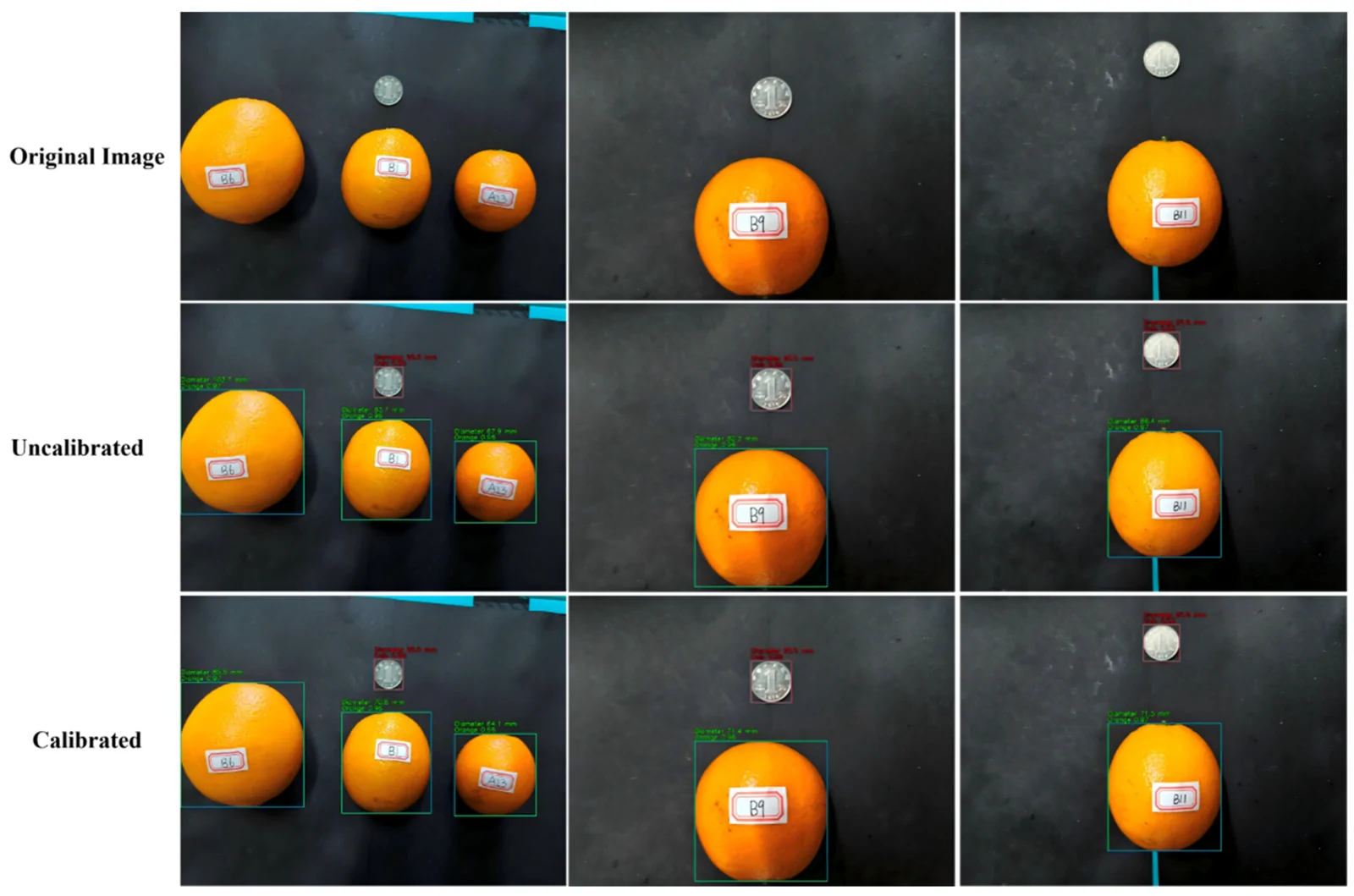
Comparison of citrus diameter estimation before and after calibration.

After calibration using the quadratic polynomial, the errors for all size groups were effectively compressed to within ±4 mm, and the error distribution approached a zero mean. As shown in Figure 20, compared with existing diameter estimation methods based on depth cameras or LiDAR, the proposed strategy requires only ordinary RGB images without specialized sensors, and the entire process can run in real time on edge devices. In summary, the two-step estimation and regression calibration framework proposed in this study reduces the MAE from 14.28 mm to 2.15 mm, validating its feasibility for achieving high-precision, low-cost diameter estimation in factory automated sorting scenarios.

### 3.7. Edge Deployment and Real-Time Validation

**Figure 21:**
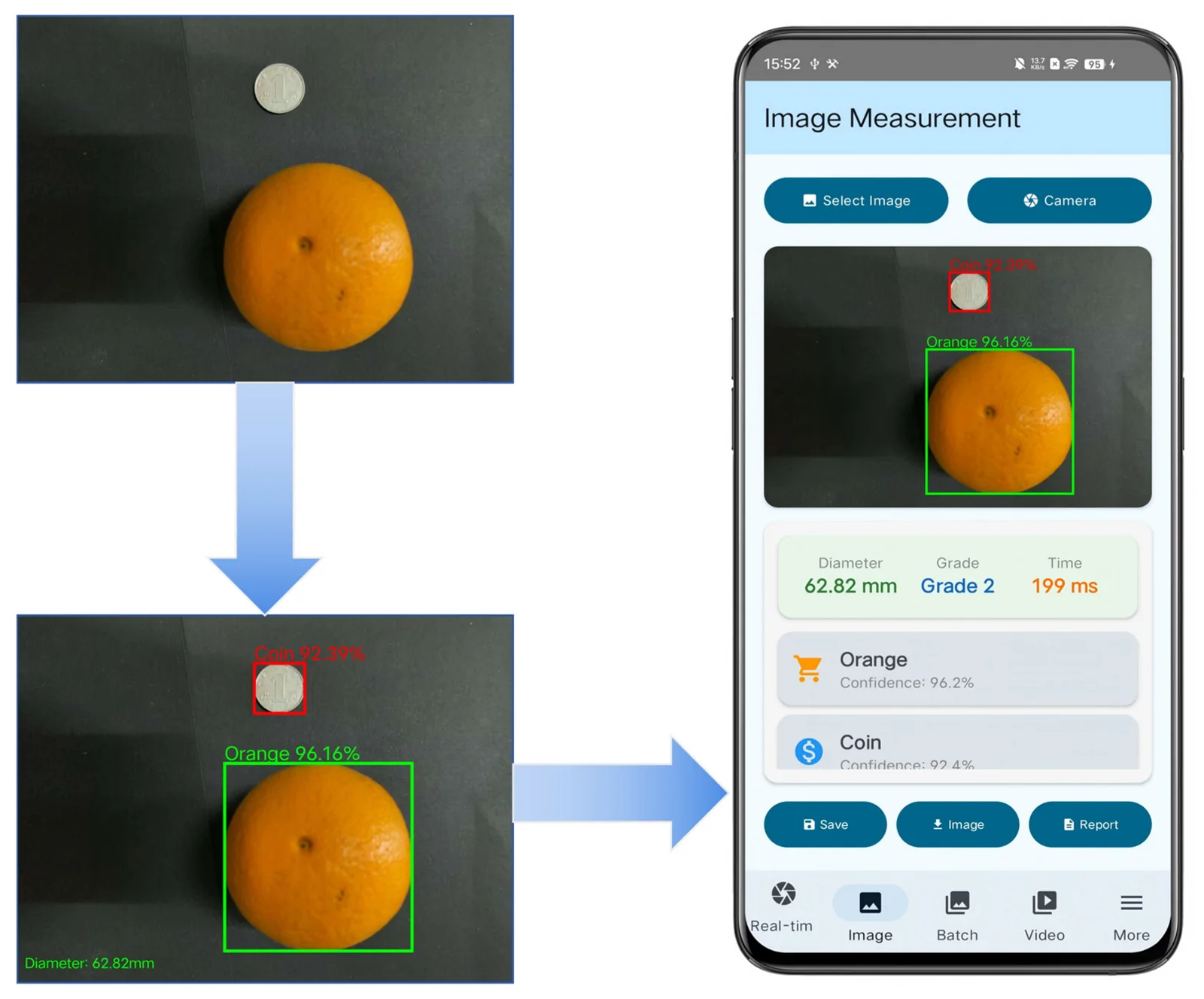
Deployment of PMDS-YOLO on Android mobile device for real-time citrus detection and diameter estimation.

To validate the feasibility and deployment effectiveness of the proposed method in real production scenarios, the trained PMDS-YOLO model was deployed on an Android mobile device (iQOO Z1X, equipped with a Snapdragon 765G processor, Adreno 620 GPU, and 8 GB RAM, running Android 11), and a citrus diameter intelligent estimation application was developed. To adapt to the edge-side inference environment, the PyTorch weight model was converted to the TensorFlow Lite format. The application was developed using Android Studio, utilizing the device’s rear camera to detect citrus fruits and coins in real time. The initial estimated diameter of the fruit was calculated based on the principle of similar triangles, and a quadratic polynomial regression model was introduced to correct the estimation error. Referring to the grading criteria shown in Table 8 (DB5304/T 003-2019), grading thresholds were defined based on fruit transverse diameter: diameter ≥70 mm as premium grade, ≥65 mm as Grade I, ≥60 mm as Grade II, and <60 mm as substandard fruit [42]. After completing the diameter estimation, the mobile application automatically matched the corresponding fruit grade, achieving an integrated edge-side workflow of object detection–diameter estimation–error calibration–intelligent grading. As shown in Figure 21, the measured end-to-end total latency was approximately 200 ms per frame, including approximately 33 ms for image acquisition, 15 ms for preprocessing (resizing to 640 × 640), 120 ms for TFLite inference, and 32 ms for post-processing (diameter calculation and calibration). On the resource-constrained Android device, the TFLite inference speed was approximately 8 FPS, with an end-to-end throughput of approximately 5 frames per second.

### 3.8. Grading Performance Evaluation

To evaluate the impact of diameter estimation errors on actual grading, the estimated and measured grades of all 100 samples were compared based on the grading standards shown in Table 8. As presented in Table 9, the calibrated model achieved an overall grading accuracy of 57.0%. The primary misclassifications were concentrated in the 60–70 mm diameter range: Premium-grade (≥70 mm) accuracy was 68.0% (17/25), Grade I (65–70 mm) accuracy was 66.0% (31/47), Grade II (60–65 mm) accuracy was 34.6% (9/26), and Substandard (<60 mm) accuracy was 50.0% (1/2). The most significant cross-boundary confusion occurred between Grade I and Grade II.

These results reveal the non-equivalence between regression accuracy optimization and grading performance improvement: even with an MAE of 2.15 mm, when grading thresholds are spaced at only 5 mm intervals, estimation errors near the boundaries can still cross the grading thresholds, leading to misclassifications. This finding carries important methodological implications: for applications targeting automated grading, minimizing MAE alone should not be the optimization objective; instead, grading-specific metrics such as grading accuracy should be directly incorporated into model optimization, or grading-oriented calibration strategies should be adopted.

To elucidate the impact of measurement errors on grading decisions, this study analyzed the distribution of estimation errors near grading boundaries. When the measured diameter falls close to a threshold (60, 65, or 70 mm), the sign of the error determines the misclassification direction: taking the 65 mm boundary as an example, a 66 mm (Grade I) fruit if underestimated would be misclassified as Grade II, while a 64 mm (Grade II) fruit if overestimated would be misclassified as Grade I. In the present dataset, Grade II (60–65 mm) fruits achieved only 34.6% accuracy, with 16 samples misclassified as Grade I, indicating that systematic positive bias dominated the misclassification direction in this size range. These results demonstrate that the risk of boundary misclassification depends not only on error magnitude but also on error direction and the fruit’s position relative to the threshold.

## 4. Discussion

This study addresses the practical requirements of citrus automated size-based grading and edge deployment. Using YOLOv13n as the baseline, a lightweight object detection model, PMDS-YOLO, was designed, and an integrated technical framework covering “object detection–scale estimation–regression calibration–intelligent grading” was constructed for non-destructive quality evaluation in postharvest processing. Through multiple sets of comparative experiments, ablation experiments, attention mechanism validation, feature extraction module comparisons, and edge deployment tests, the effectiveness of the model structure improvements, diameter estimation method, and engineering deployment strategy was systematically validated, while the limitations of the current approach in terms of scene adaptability, method robustness, and system scalability were also identified.

In terms of the effectiveness of model improvements, the lightweight strategy proposed in this study exhibits clear problem orientation. Specifically, compressing the width factor to 0.125 reduces parameters by 76.4%, drastically cutting redundant parameters while preserving core feature representation capability. Replacing the backbone network with MCSR-MobileOne achieves a 28.86% reduction in parameters and a 0.38-percentage-point gain in mAP@0.5:0.95. It significantly enhances the model’s ability to discriminate multi-scale citrus targets from complex backgrounds with reduced computational overhead. Introducing DySnakeConv to refine the DSC3k2 module yields a 6.91% reduction in parameters together with a 0.78-percentage-point increase in mAP@0.5:0.95, verifying its improved adaptability to irregular fruit contours. Adopting Soft-NMS to replace conventional NMS effectively alleviates missed detections in dense-object scenarios without altering parameter count and computational cost. Benefiting from the synergy of these four improvements, the proposed model reaches an mAP@0.5:0.95 of 86.57%, corresponding to a 0.99-percentage-point improvement over the baseline, despite a 76.4% reduction in total parameters. These findings validate the scientific validity of the model-structure optimization direction and confirm its suitability for resource-constrained food-processing environments.

In terms of diameter estimation, this study employs a 25 mm standard coin as a scale reference. By utilizing the ratio of the bounding box heights of the fruit and the coin, a low-cost initial diameter estimation is achieved based on the principle of similar triangles, reducing reliance on specialized equipment such as depth cameras and LiDAR. Compared with traditional methods relying on complete contours (e.g., ellipse fitting and contour detection), the proposed method estimates diameter from bounding boxes without requiring intact fruit contours, offering greater robustness to occlusion and complex backgrounds. Traditional methods may achieve higher accuracy when contours are clear and regular, though such conditions are difficult to consistently satisfy in natural orchard environments. The geometric assumption of this method is that the coin and fruit are approximately coplanar, and the height direction of the bounding box is parallel to the image plane. Nevertheless, in practical scenarios, perspective distortion, irregular fruit contours, partial occlusion of the coin by branches and leaves, different depth planes between the coin and fruit, and tilted shooting angles make this assumption difficult to satisfy. Under such circumstances, the bounding-box height cannot accurately reflect the physical size, and the proportional relationship derived from similar triangles is distorted, resulting in considerable systematic bias in the initial estimation results. These factors go beyond the correction capability of quadratic polynomial regression and constitute the main limitations of the proposed method in practical deployment. Although segmentation-based approaches could theoretically yield more accurate fruit contours, their high annotation cost and slow inference speed make them impractical for real-time sorting applications. Therefore, this study adopts the bounding box-based scheme and employs subsequent regression calibration to compensate for its inherent systematic bias. Among the various regression models compared, quadratic polynomial regression was found to eliminate the aforementioned errors to the greatest extent, reducing the mean absolute error of diameter estimation from 14.28 mm to 2.15 mm. The overall grading accuracy reached 57.0%, among which Grade II fruits (60–65 mm) achieved an accuracy of only 34.6%, with most being misclassified as Grade I. This result reveals the essential difference between regression accuracy and grading performance: MAE measures the average deviation on a continuous scale, whereas grading accuracy measures classification correctness at discrete thresholds. When the grading interval is as narrow as 5 mm, even minor estimation errors near the boundaries can easily lead to cross-grade misclassification. Future work should therefore focus on grading-oriented optimization strategies, such as boundary-aware loss functions or direct optimization of grading accuracy, rather than solely minimizing regression errors.

From the perspective of food quality assurance, the proposed method offers several advantages over conventional grading approaches. First, it provides objective and reproducible diameter measurements, eliminating the subjectivity inherent in manual visual inspection and ensuring consistent grading outcomes. Second, the non-destructive nature of the method preserves fruit integrity, which facilitates intact postharvest fruit management and provides technical potential for reducing unnecessary fruit loss during industrial sorting. Third, the low-cost, edge-deployable design makes the technology accessible to small- and medium-sized citrus processing facilities, democratizing access to automated quality sorting technologies that were previously available only to large-scale operations.

This study has certain limitations. First, the dataset originates from a single variety in a single production area, with insufficient coverage of illumination conditions, occlusion levels, and background complexity; the model’s generalization capability across different varieties, regions, extreme weather, and complex field environments remains to be validated. Second, the diameter estimation method hinges on the premise that the coin and fruit appear in the same frame and are approximately coplanar; deviations in shooting angle, distance, or reference object placement can substantially amplify estimation errors. While quadratic polynomial regression effectively mitigates systematic bias within the calibrated range, its corrective capacity diminishes under shooting conditions that deviate substantially from this range. Third, in cases of highly dense fruits, severe occlusion, or extremely small target scales, the model exhibits a small number of missed detections and decreased confidence, indicating that small-target perception and anti-interference capability require further enhancement. Fourth, in actual industrial scenarios, system throughput is influenced by multiple factors including conveyor belt speed, fruit density, camera configuration, and multi-fruit parallel processing strategies, and the end-to-end latency of approximately 200 ms per frame remains a bottleneck for high-density, high-speed industrial sorting lines. Fifth, this study focuses exclusively on diameter as the grading criterion, whereas comprehensive fruit quality assessment in the food industry also considers other attributes such as color, sugar content, acidity, and internal defects, which are essential for determining overall fruit quality and consumer acceptance.

To address the above limitations, future research can focus on the following directions: First, expand the citrus dataset to cover multiple varieties, scenarios, and lighting conditions, thereby improving the model’s generalization capability and environmental adaptability. Second, introduce camera calibration, perspective correction, and monocular depth estimation methods to reduce the strong dependence of diameter estimation on the coplanarity and same-frame requirements of the reference object, and expand the coverage of the calibration set, thereby improving estimation accuracy and robustness. Third, optimize the small-target detection head and feature fusion structure to enhance detection stability in densely occluded scenarios. Fourth, to meet the demands of industrial-scale high-throughput sorting, adopt model quantization (INT8), dedicated edge AI accelerators, and multi-camera parallel processing architectures to improve system throughput, with validation on actual sorting lines. Fifth, extend the current framework to incorporate multi-modal quality attributes—such as color, sugar content, and internal defects—through the integration of spectral imaging or hyperspectral techniques, enabling comprehensive, multi-parameter fruit quality evaluation that aligns with the full scope of food quality assessment in the citrus industry.

## 5. Conclusions

To address the issues of large parameter counts, high computational costs, and difficulty in deploying existing citrus detection models on edge devices, as well as the reliance on specialized equipment, high costs, and low efficiency of traditional diameter measurement methods, this study takes YOLOv13n as the baseline, proposes an ultra-lightweight detection model PMDS-YOLO, and establishes a citrus diameter estimation and intelligent grading method suitable for edge deployment for non-destructive food quality assessment in postharvest processing. Through lightweight model structure improvements, diameter estimation and regression calibration, end-to-end system integration, and edge deployment validation, the main conclusions are drawn as follows:

1. In this study, the PMDS-YOLO model was constructed through width factor compression, MCSR-MobileOne backbone replacement, DSC3k2 module improvement with dynamic snake convolution, and Soft-NMS optimization, achieving a favorable balance between lightweight design and detection accuracy. The model achieves a parameter count of 0.58 M, a model size of only 1.44 MB, a computational cost of 2.3 G, and an inference frame rate of 48.0 FPS. The mAP@0.5:0.95 reaches 86.41%, and the precision reaches 97.43%. The proposed method achieves competitive overall performance relative to mainstream lightweight YOLO models, demonstrating its deployment potential on resource-constrained edge devices and providing a feasible technical solution for postharvest quality assessment of the tested citrus variety.
2. A low-cost diameter estimation scheme of “coin scale reference–similar triangle estimation–quadratic polynomial regression calibration” is proposed, which achieves high-precision diameter measurement without the need for depth sensors. After calibration, the mean absolute error is reduced to 2.15 mm, the root mean square error is 3.16 mm, and the coefficient of determination R^2^ reaches 0.85. The achieved measurement accuracy indicates that the proposed method holds practical promise for citrus automated grading, although further optimization is required to enhance grading-specific performance.
3. An end-to-end intelligent grading system from object detection, diameter calculation, and error calibration to grade determination is constructed and successfully deployed on Android mobile devices, supporting offline, real-time non-destructive citrus detection and grading.
4. The lightweight improvement strategy, low-cost diameter measurement method, and edge deployment scheme proposed in this study can serve as a referable technical paradigm for lightweight detection of fruit and vegetable targets, visual size estimation, and agricultural edge intelligence applications. More broadly, this work contributes to the advancement of non-destructive food quality assessment technologies, with significant implications for improving product consistency, reducing postharvest losses, and enhancing supply chain efficiency in the citrus industry.

In summary, PMDS-YOLO achieves a favorable balance among extreme lightweightness, detection accuracy, and engineering practicality. By enabling objective, consistent, and high-throughput fruit size grading, the proposed system provides a practical, low-cost, and edge-deployable solution for non-destructive food quality assessment in citrus postharvest processing, offering promising prospects for industrial adoption and commercialization.

## Figures and Tables

**Table 1:** Main training configurations of compared models.

Model	Optimizer	Initial LR	LR Schedule	Epochs	Size
FasterRcnn	SGD	0.005	StepLR (3, 0.33)	100	640
SSD	SGD	0.0005	Step (5, 0.3)	100	640
RetinaNet	SGD	0.005	Step (5, 0.5)	100	640
YOLO-series	SGD	0.05	Cosine	200	640

**Table 2:** Comparative experimental results of different object detection models.

Model	mAP0.50:0.95 (%)	mAP0.75 (%)	P (%)	R (%)	Parameters (M)	Model Size (MB)
FasterRcnn	71.14	79.08	89.35	77.64	82.36	628.56
SSD	75.69	82.85	90.96	78.53	13.19	101.20
RetinaNet	82.59	89.13	96.24	92.11	32.22	245.35
YOLOv8n	85.79	90.28	95.53	92.27	3.01	5.98
YOLOv10n	85.45	90.11	96.38	89.87	2.70	5.52
YOLOv11n	86.04	90.43	95.31	92.73	2.59	5.25
YOLOv12n	85.71	89.42	94.28	91.85	2.52	5.23
YOLOv13n	85.42	89.77	95.35	91.25	2.46	5.20
YOLOv26n	84.01	88.78	95.99	90.02	2.50	5.17
PMDS-YOLO	86.41	90.84	97.43	92.42	0.58	1.44

**Table 3:** Ablation experimental results of the PMDS‑YOLO model.

Model	Pico	MobilOne-MCSR	DySnakeConv	Soft_nms	mAP0.50:0.95 (%)	P (%)	Parameters (M)	FLOPs (G)
YOLOv13	-	-	-	-	85.42	95.35	2.46	6.4
YOLOv13p	√	-	-	-	84.52	95.04	0.81	2.5
Model2	-	√	-	-	85.80	95.30	1.75	5.8
Model3	-	-	√	-	86.20	95.21	2.29	6.1
Model4	√	√	-	-	84.18	95.69	0.63	2.4
Model5	√	-	√	-	84.50	95.07	0.76	2.4
Model6	-	√	√	-	85.53	96.16	1.48	5.3
Model7	√	√	√	-	84.77	95.93	0.58	2.3
Model8	√	√	√	√	86.41	97.43	0.58	2.3

Note: “√” indicates that the corresponding module is adopted, and “-” indicates that the corresponding module is not adopted.

**Table 4:** Performance comparison of different attention mechanisms.

Model	mAP0.50:0.95 (%)	mAP0.50 (%)	P (%)	Parameters (M)	FLOPs
PMD	83.60	95.08	94.14	0.57	2.1
+MCSR	84.77	95.39	95.93	0.58	2.3
+CA	84.19	95.34	94.50	0.57	2.2
+CBAM	83.56	94.53	95.82	0.57	2.2
+EMA	84.46	95.17	94.04	0.57	2.3
+GAM	83.85	95.01	95.15	0.58	2.2

**Table 5:** Performance comparison of different DSC3k2 variants.

Model	mAP0.50:0.95 (%)	mAP0.50 (%)	P (%)	R	Parameters (M)
YOLOv13n	85.42	96.06	95.35	91.25	2.46
yolov13-DSC3k2_DySnakeConv	86.20	96.38	95.21	92.39	2.29
yolov13-DSC3k2_BiFormer	85.19	96.00	96.04	91.94	2.53
yolov13-DSC3k2_GnConv	85.06	95.88	94.57	91.65	2.56
yolov13-DSC3k2_iRMB	85.24	95.86	95.51	91.24	2.53
yolov13-DSC3k2_RFAConv	85.84	96.03	95.64	91.11	2.84

**Table 6:** Performance comparison between PMDS-YOLO and the baseline model.

Model	Random Seed	mAP0.50:0.95 (%)	P (%)	Parameters (M)	Model Size (MB)	FPS
YOLOv13n	0	85.42	95.35	2.46	5.20	28.8
	1	85.14	94.92	2.46	5.20	28.8
	2	84.98	95.01	2.46	5.20	28.8
	42	85.44	95.11	2.46	5.20	28.8
	Mean ± Std	85.25 ± 0.21	95.10 ± 0.19	2.46	5.20	28.8
PMDS-YOLO	0	86.41	97.43	0.58	1.44	48.0
	1	86.16	96.53	0.58	1.44	48.0
	2	85.88	96.76	0.58	1.44	48.0
	42	86.43	97.38	0.58	1.44	48.0
	Mean ± Std	86.22 ± 0.24	97.03 ± 0.42	0.58	1.44	48.0

**Table 7:** Performance comparison of different regression models for diameter calibration.

Model	MAE (mm)	RMSE (mm)	R^2^
Linear Regression (with intercept)	2.84	3.56	0.81
Linear Regression (through the origin)	3.54	4.07	0.76
Quadratic Polynomial	2.15	3.16	0.85
Logarithmic Regression	2.97	3.91	0.78
Ridge Regression	2.84	3.55	0.81

**Table 8:** Grading standard for citrus fruit based on diameter.

Diameter Range	Grade	Commercial Value
≥70 mm	Premium	Highest (Premium Fruit)
65–70 mm	Grade I	Medium-High
60–65 mm	Grade II	Standard Qualified
<60 mm	Substandard	Low Price/Processing

**Table 9:** Grading confusion matrix.

True Grade	Premium	Grade I	Grade II	Substandard	Accuracy (%)
Premium	17	8	0	0	68.0
Grade I	7	31	9	0	66.0
Grade II	1	16	9	0	34.6
Substandard	0	0	1	1	50.0

## Data Availability

The original citrus fruit image dataset and manual fruit measurement data presented in the study are openly available in ScienceDB at https://doi.org/10.57760/sciencedb.010ur. Partial source code of the PMDS-YOLO model (version 1.0) is available on GitHub (https://github.com/yolo403/PMDS-YOLO-.git accessed on 7 September 2026).

## Abbreviations

The following abbreviations are used in this manuscript:

MCSR	Multi-scale Channel–Spatial Residual attention module
DySnakeConv	Dynamic snake convolution
Soft-NMS	Soft non-maximum suppression
DWConv	Depthwise convolution
DDConv	Depthwise dilated convolution
ECA	Efficient channel attention
SA	Spatial attention
